# A longitudinal Analysis of the Association between Socioeconomic Position and Multimorbidity in the European Prospective Investigation into Cancer and Nutrition Study

**DOI:** 10.14336/AD.2024.1166

**Published:** 2025-12-03

**Authors:** Luca Manfredi, Barbara Sodano, Chiara Raganato, Federica Buscema, Lorenzo Milani, Alberto Catalano, Heinz Freisling, Pietro Ferrari, Alem Abraha, Rudolf Kaaks, Verena Katzke, Salvatore Panico, Christian Skødt Antoniussen, Christina C. Dahm, Sandar Tin Tin, Roel Vermeulen, Ilonca Vaartjes, Anne Tjønneland, Anja Olsen, Sandra Colorado-Yohar, Sara Grioni, Marc J. Gunter, Matthias B. Schulze, Reynalda Cordova, Maria-Jose Sánchez, Catalina Bonet Bonet, Rosario Tumino, Olatz Mokoroa, Giovanna Masala, Marcela Guevara Eslava, Monique Verschuren, Carlotta Sacerdote, Fulvio Ricceri

**Affiliations:** ^1^Centre for Biostatistics, Epidemiology, and Public Health, Department of Clinical and Biological Sciences, University of Turin, Orbassano, Turin, Italy; ^2^Department of Statistics, Computer Science, Applications, University of Florence, Florence, Italy; ^3^University of Turin, Turin, Italy; ^4^Department of Translational Medicine, University of Piemonte Orientale, Novara, Italy; ^5^International Agency for Research on Cancer (IARC-WHO), Lyon, France; ^6^Division of Cancer Epidemiology, German Cancer Research Center (DKFZ), Heidelberg, Germany; ^7^Dipartimento di Medicina Clinica e Chirurgia, School of Medicine, Federico II University, Naples, Italy; ^8^Department of Public Health, Aarhus University, Aarhus, Denmark; ^9^Nuffield Department of Population Health (NDPH), University of Oxford, Oxford, England; ^10^Institute for Risk Assessment Sciences, Utrecht University, Utrecht, The Netherlands; ^11^Julius Center for Health Sciences and Primary Care, University Medical Center Utrecht, Utrecht, The Netherlands; ^12^Danish Cancer Institute, Copenhagen, Denmark; ^13^Department of Public Health, University of Copenhagen, Copenhagen, Denmark; ^14^Department of Epidemiology, Murcia Regional Health Council, IMIB-Arrixaca, Murcia, Spain; ^15^CIBER Epidemiología y Salud Publica (CIBERESP), Madrid, Spain;; ^16^Research Group on Demography and Health, National Faculty of Public Health, University of Antioquia, Medellín, Colombia.; ^16^Epidemiology and Prevention Unit, Fondazione IRCCS Istituto Nazionale dei Tumori di Milano, Milan, Italy;; ^18^School of Public Health, Imperial College London, London, UK; ^19^Department of Molecular Epidemiology, German Institute of Human Nutrition Potsdam-Rehbruecke, Nuthetal, Germany; ^20^Institute of Nutritional Science, University of Potsdam, Nuthetal, Germany; ^21^Faculty of Life Sciences, Department of Nutritional Sciences, University of Vienna, Vienna, Austria; ^22^Escuela Andaluza de Salud Pública (EASP), 18011 Granada, Spain;; ^23^Instituto de Investigación Biosanitaria ibs.GRANADA, 18012 Granada, Spain;; ^24^Unit of Nutrition and Cancer, Catalan Institute of Oncology - ICO, L'Hospitalet de Llobregat, Spain; ^25^Nutrition and Cancer Group; Epidemiology, Public Health, Cancer Prevention and Palliative Care Program; Bellvitge Biomedical Research Institute - IDIBELL, L'Hospitalet de Llobregat, Spain; ^26^Hyblean Association for Epidemiology Research, AIRE ONLUS Ragusa, Ragusa, Italy; ^27^Ministry of Health of the Basque Government, Sub Directorate for Public Health and Addictions of Gipuzkoa, San Sebastián, Spain;; ^28^BioGipuzkoa (BioDonostia) Health Research Institute, Epidemiology of Chronic and Communicable Diseases Group, San Sebastián, Spain;; ^29^Institute for Cancer Research, Prevention and Clinical Network (ISPRO), Florence, Italy; ^30^Instituto de Salud Pública y Laboral de Navarra, 31003 Pamplona, Spain;; ^31^Navarra Institute for Health Research (IdiSNA), 31008 Pamplona, Spain; ^32^Julius Center for Health Sciences and Primary Care (ELT, MK, WMMV, MIG), University Medical Center Utrecht and Utrecht University, Utrecht, The Netherlands; ^33^National Institute for Public Health and the Environment (ACJN, WMMV), Bilthoven, The Netherlands; ^34^Department of Health Sciences, University of Eastern Piedmont, 28100 Novara, Italy.

**Keywords:** multimorbidity, inequalities, lifestyle, social epidemiology

## Abstract

The association between socioeconomic position (SEP) and non-communicable diseases (NCDs) is well established, but its role in driving multimorbidity remains unclear. Multimorbidity, defined as the co-occurrence of more than one chronic condition, is linked to higher mortality and reduced quality of life. This study investigates the association between SEP and multimorbidity using data from the European Prospective Investigation into Cancer and Nutrition (EPIC). Incident cases of cancer, type 2 diabetes (T2D), and cardiovascular diseases (CVDs) were analysed alongside lifestyle factors such as smoking status, alcohol intake, body mass index (BMI), physical activity and diet. Multimorbidity was defined as the presence of at least two of the studied NCDs. SEP was assessed using the Relative Index of Inequality (RII) and categorized into high, medium, and low SEP. The cohort included 277 302 participants (60.7% women) from seven countries, enrolled between 1992-2000 and followed until the first diagnosis, end of follow-up (31/12/2007), or death. For transitions to multimorbidity, follow-up extended from the first diagnosis to the second diagnosis, end of follow-up, or death. Multistate models were used to examine the nine possible transitions to first diagnoses and multimorbidity combinations. Lifestyle factors were risk factors for all the transitions, except alcohol intake. In the main model, not stratified by sex, low SEP was associated with higher risks of progressing from cancer to CVD (Hazard Ratio (HR): 1.23, CI: 1.02-1.50), CVD to T2D (HR: 1.35, CI: 1.07-1.71), and cancer to T2D (HR: 1.37, CI: 1.10-1.69). These findings highlight the persistent influence of social inequalities on the risk of multimorbidity, even in individuals with an existing chronic condition.

## INTRODUCTION

In the last decades, the rise in life expectancy and the consequent population aging (*Life expectancy at birth fell to 80.1 years in 2021*. Eurostat News; https://ec.europa. eu/eurostat/web/products-eurostat-news/w/DDN-20230316-1. Accessed 2 Jun 2023) [1] have brought with them an increasing burden of non-communicable diseases (NCDs), that is estimated to be responsible for the vast majority of avoidable premature deaths. Indeed, as people live longer, the prevalence of chronic conditions has been steadily growing. Consequently, the health management issues and the financial impact on the society are expected to grow in the next decades (*Non communicable diseases*. WHO; www.who.int/news-room/fact-sheets/detail/noncommunicable-diseases. Accessed 2 Jun 2023. (2023). *Overview.* European Commission; https://health.ec.europa.eu/non-communicable-diseases/overview_en. Accessed 2 Jun 2023) [2].

A particular vulnerable subset of the population is the group of patients living with multimorbidity, which has complex care needs that imply a greater healthcare use, to cope with the persistent chronic conditions (2023). *Overview.* European Commission; https://health.ec. europa.eu/non-communicable-diseases/overview_en. Accessed 2 Jun 2023) [3, 4]. Multimorbidity, usually defined as the co-occurrence of more than one chronic condition [5] is not just the sum of different diseases, but involves possible synergies and interactions among diseases [6], and is associated with higher mortality and reduced quality of life [3, 7]. Although a strong association exists between aging and multimorbidity, others factors are involved, such as sex [8, 9], ethnicity [10], lifestyle [11-13] and environmental factors [14], as determinants of underlying mechanisms leading to multimorbidity [2]. Different studies have also shown a strong relationship between SEP and multimorbidity. However, the importance of SEP in driving multimorbidity is not yet fully understood [7, 15, 16] and the majority of studies are cross-sectional, thus not suited for establishing temporality [2, 16, 17]. Furthermore, the relation between SEP and multimorbidity is complex and reverse causality has to be considered when looking at the results. In addition, inequalities in aging can be driven by an earlier onset of morbidity during lifetime in people with low SEP, leading to faster trajectories towards multimorbidity [18]. Due to the high complexity of this relationship, accounting for temporality is paramount.

As a related issue, following the definition of Krieger et al. (1997) [19], SEP is not directly measurable. Different definitions are present in the literature, that do not overlap completely [2, 20]. In the present work the Relative Index of Inequality (RII), based on the educational level, was considered as a proxy of SEP. The educational level has numerous advantages: it is established early in life and generally remains consistent beyond around age 30, reducing the possibility of reverse causality, in contrast to indicators like income, which can fluctuate significantly over time. The educational level can capture the family and cultural background of an individual, acting as a proxy of awareness in terms of health issues and maintenance as well as the ability to access to healthcare system, in addition to being correlated with the income level in adulthood [17, 19].

The present study aims at assessing the magnitude of the association between SEP and multimorbidity in order to shed light on the social inequalities that are in place in the context of an aging population. Using data from one of the largest cohort studies in Europe allows the consideration of both lifestyle and sociodemographic risk factors in a longitudinal setting, to disentangle the independent effect of SEP on multimorbidity. The longitudinal nature of the study is crucial for establishing temporality and understanding the evolving relationship between SEP and multimorbidity over time.

## METHODS

### Setting and study population

The present study exploited data collected from the European Prospective Investigation into Cancer and Nutrition (EPIC) study. EPIC is one of the largest cohort studies in Europe and encompasses information about diet, lifestyle, medical history and anthropometric measurements. Data was gathered in the period 1992-2000 from 23 centres in 10 European countries (Italy, France, Spain, Greece, United Kingdom, Netherlands, Germany, Norway, Sweden and Denmark) [21]. The population under investigation is the result of sampling on voluntary basis across all the European centres. Enrolled subjects were required to fill out two questionnaires, about diet intake and lifestyle, respectively. Moreover, a visit into a reference centre was conducted in the majority of centres, in order to obtain anthropometric measurements and biological samples.

Subjects who presented at least one disease among cancer, myocardial infarction, angina, stroke, type 2 diabetes (T2D) at baseline were excluded. Moreover, subjects with missing information on education at baseline were excluded (Supplementary Fig. 1). Finally, since Norway and Greece did not have information on T2D cases and France did not record cardiovascular diseases (CVD) cases, data from those centres was excluded from the present work. The final sample included 277 302 subjects.

More details on the study population, data collection, study design and measurements are available from previous works [22-25].

### Exposure and covariates

To study the primary variable of interest, SEP, the Relative Index of Inequality (RII) was considered. RII is a standardized index used as a proxy of SEP, that allows to compare participants from different countries and cohorts of birth. In particular, the index was built by assigning it a value based on a classification of five educational levels (no qualifications, completed primary school, completed technical school, high school diploma, post-secondary or university education), according to the proportion of participants within the strata formed by each country, by 10-year age group, and by gender. To calculate the RII score, the midpoint of the cumulative proportional distributions of each educational level was then used [26, 27]. Subsequently, the RII was divided into three categories, using the distribution tertiles: RII = 1 (High SEP), RII = 2 (Medium SEP), RII = 3 (Low SEP).

In regards to lifestyle variables, data on smoking status at baseline was used. Height and weight were utilized to calculate the body mass index (BMI). Physical activity levels were calculated according to the Cambridge Index.[24] Dietary and alcohol intake information was drawn by centre specific questionnaires, based on the last 12 months dietary habits. The adherence to a healthy diet was evaluated using a modified Mediterranean dietary score (mrMDS) [25, 28].

The variables have been categorized as follows: smoking status (never, former, current smoker); BMI < 25, 25 <= BMI < 30, BMI >= 30; physical activity, according to the Cambridge physical activity Index (Pa index: inactive, moderately inactive, moderately active, active); alcohol intake at baseline (men: < 1, 1-24, > 24 g/day; women: < 1, 1-12, >12 g/day); mrMDS (low, medium, high).

Finally, age at enrolment and enrolment centre were also considered.

### Outcomes of interest

Deaths were collected by the enrolment centres through the mortality registries [21].

Cancer data was codified by the International Classification of Diseases for Oncology, 3rd Edition (ICD-O-3) (*International Classification of Diseases for Oncology, 3rd Edition (ICD-O-3).* WHO; www.who.int/standards/classifications/other-classifications/international-classification-of-diseases-for-oncology. Accessed 6 Jun 2023).

Data on T2D and CVDs were gathered from two case-cohort studies nested within the EPIC study: EPIC-InterAct was a study conducted between 1992 and 2007, with the main goal of investigating the association between genetic factors, lifestyle and T2D. The InterAct consortium followed a high sensitivity approach to identify and verify all incident T2D cases in the EPIC cohort. For each potential case evidence was sought from at least two independent sources, by reviewing the EPIC datasets at each involved centre [23]. EPIC-CVD, active in the period 1992-2010, investigated the aetiology of CVDs. It exploited data on cause-specific mortality collected within the EPIC study. Furthermore, incident non-fatal CVD events were recorded across all centres, using different methods of ascertainment. To account for these differences, validation studies have been conducted to assess the accuracy of these non-fatal coronary outcomes [22]. Norway and Greece did not participate in the EPIC-Interact study and France was not involved in the EPIC-CVD study.

CVD events were classified according to the International Classification of Diseases: codes 410-414 and 430-438 from the 9^th^ Edition (ICD-9), I20-I25 from the10^th^ Edition (ICD-10) [22, 29]. Each event was classified as fatal or not (except for angina, I20, since it is a non-fatal event by definition).

### Statistical analyses

To give a detailed overview of the studied diseases and their possible connections into multimorbidity clusters, we conducted a preliminary descriptive analysis of subjects with multimorbidity, using Multiple Correspondence Analysis (MCA) and cluster analysis. For this analysis, CVDs were split into coronary heart diseases (CHD) and stroke. Moreover, morphology, diagnosis date, site and staging codes for cancer cases were retrieved. The recoding led to the identification of 25 types of cancers or cancer groups: mouth, leukaemia, cervix, colorectal, body of the uterus, duodenum, brain, oesophagus, liver, larynx, lymphoma, breast, melanoma, ovary, pancreas, lung, prostate, kidney, stomach, soft tissues, thyroid, bladder, other digestive, other female genitals, and other cancers. For the analysis, conducted separately for two datasets divided by sex, the cancers were grouped based on the body system and the similarity of risk factors into new dichotomous variables, each indicating the presence (or absence) of the considered cancer (or group of cancers) (see supplementary material for details). In the men's dataset, this grouping led to the identification of 10 cancer groups, corresponding to 10 different dichotomous variables (mouth and thyroid, colorectal, liver and pancreas, leukaemia and lymphoma, melanoma, bladder, lung, digestive tract, bladder and kidney, other cancers), while in the women's dataset, 13 groups were identified (mouth and thyroid, cervix, colorectal, body of the uterus, liver and pancreas, leukaemia and lymphoma, breast, melanoma, other female genital, lung, digestive tract, bladder and kidney, other cancers).

To examine the transitions between baseline (disease-free state) and the first diagnosis, the participants were followed from the date of enrolment to one of the following events: date of first diagnosis (cancer, T2D or CVD), end of follow up date (31/12/2007), date of death. Similarly, to study the transition to multimorbidity as the endpoint, the follow up (in years) encompassed the time from the date of the first diagnosis to one of the following events: date of second diagnosis, end of follow up date (31/12/2007), date of death. Longitudinal data have been analysed employing multistate models, based on Cox regression analyses [30-34]: survival data measure the time interval from the start of the study to a certain event. If different types of events are possible, a model that describes the progression towards each event is needed. Multistate models generalize competing risk models by describing transitions between intermediate states. These models are useful to describe a stochastic process, in which every subject is within one of the possible discrete states. In this context, events are the transitions between states. All the 9 possible transitions across different states and their associations with SEP, were analysed: from disease-free state to one of the three diseases considered, and from the first diagnosis to all possible multimorbidity combinations of two diseases.

The main model encompassed the whole sample, taking sex and enrolment centre as stratification variables, and adjusting for age, BMI, smoking status, mrMDS, physical activity and alcohol intake. Stratification enables the model to have different baseline hazards for each level of the stratification variables, accounting for possible different baseline risks in different centres and across sexes. Two additional secondary models were considered by dividing the sample by sex. These models are specified identically to the main model, with the exception of the sex variable. This approach aims at simplifying results interpretation and at highlighting potential differences between sexes.

Finally, a graphical comparison was generated to visualize the transitions to a first diagnosis and from the first diagnosis to multimorbidity according to SEP: the transition probabilities were calculated for each tertile of the RII variable, to better understand the probability to belong to a particular state, having a certain level of SEP, during the entire follow up.

Analyses were conducted in R (Version: 4.3.0) (*R: The R Project for Statistical Computing.* https://www.r-project.org/. Accessed 1 Sep 2023).

**Table 1:** Descriptive characteristics of the study sample by Relative Index of Inequality (RII) tertiles.

	RII = 1		RII = 2		RII = 3	
	Male	Female	Male	Female	Male	Female
Number of participants (n)	36231	53378	33950	56883	38851	58009
Follow up (IQR)	11.98 (2.15)	12.25 (2.02)	12.30 (2.31)	12.18 (1.97)	12.15 (2.20)	12.32 (2.02)
Age at recruitment (IQR)	52.19 (12.18)	51.69 (13.59)	52.42 (11.67)	51.60 (14.31)	53.88 (12.56)	53.20 (12.56)
T2D, n (%)	1712 (4.73)	1382 (2.59)	2295 (6.76)	2137 (3.76)	2832 (7.29)	2701 (4.66)
CVD, n (%)	1927 (5.32)	1351 (2.53)	2289 (6.74)	1496 (2.63)	3048 (7.85)	2201 (3.79)
Cancer, n (%)	3755 (10.36)	4926 (9.23)	3586 (10.56)	5102 (8.97)	4648 (11.96)	5472 (9.43)
Multimorbidity, n (%)	641 (1.77)	375 (0.70)	770 (2.27)	519 (0.91)	1113 (2.86)	733 (1.26)
BMI (SD)	25.88 (3.27)	24.67 (3.99)	26.47 (3.50)	25.48 (4.31)	26.84 (3.68)	26.43 (4.61)
BMI category, n (%)						
Normal	3698 (10.2)	5167 (9.7)	4940 (14.6)	7773 (13.7)	6715 (17.3)	11366 (19.6)
Overweight	17335 (47.8)	15814 (29.6)	17052 (50.2)	19275 (33.9)	19798 (51.0)	21796 (37.6)
Obesity	15198 (41.9)	32397 (60.7)	11958 (35.2)	29835 (52.4)	12338 (31.8)	24847 (42.8)
Alcohol, g/day (SD)	22.28 (21.74)	9.91 (12.71)	21.76 (23.84)	8.67 (11.83)	20.62 (23.92)	6.77 (10.66)
Alcohol category, n (%)						
Non-drinker	1639 (4.5)	7060 (13.2)	1998 (5.9)	8160 (14.3)	2849 (7.3)	11043 (19.0)
men: 1-24 g/day; women: 1-12 g/day	16811 (46.4)	19077 (35.7)	15702 (46.3)	19856 (34.9)	17064 (43.9)	17723 (30.6)
men: > 24 g/day; women: > 12 g/day	17781 (49.1)	27241 (51.0)	16250 (47.9)	28867 (50.7)	18938 (48.7)	29243 (50.4)
Smoking Status, n (%)						
Never smoker	13365 (36.89)	27887 (52.24)	11519 (33.93)	30135 (52.98)	11119 (28.62)	31669 (54.59)
Former smoker	13611 (37.57)	14261 (26.72)	11806 (34.77)	13054 (22.95)	14514 (37.36)	11879 (20.48)
Current smoker	9056 (25.00)	11142 (20.87)	10432 (30.73)	13569 (23.85)	13003 (33.47)	14279 (24.62)
Unknown	199 (0.55)	88 (0.16)	193 (0.57)	125 (0.22)	215 (0.55)	182 (0.31)
Pa Index, n (%)						
Inactive	6348 (17.52)	10958 (20.53)	5437 (16.01)	12094 (21.26)	6195 (15.95)	16085 (27.73)
Moderately inactive	13784 (38.04)	19530 (36.58)	10166 (29.94)	19938 (35.05)	10215 (26.29)	18734 (32.29)
Moderately active	8726 (24.08)	12606 (23.62)	8791 (25.89)	12628 (22.20)	9549 (24.58)	11569 (19.94)
Active	6978 (19.26)	9898 (18.54)	9147 (26.94)	11700 (20.57)	12330 (31.74)	10936 (18.85)
Missing	395 (1.09)	386 (0.72)	409 (1.20)	523 (0.92)	562 (1.45)	685 (1.18)
mrMDS, n (%)						
Low	10968 (30.27)	11277 (21.13)	13313 (39.21)	16162 (28.41)	17338 (44.63)	17465 (30.11)
Medium	16909 (46.67)	25718 (48.18)	13618 (40.11)	26968 (47.41)	14721 (37.89)	25709 (44.32)
High	8354 (23.06)	16383 (30.69)	7019 (20.67)	13753 (24.18)	6792 (17.48)	14835 (25.57)

T2D: Type 2 Diabetes; CVD: Cardiovascular Disease; BMI, Body Mass Index: Normal = BMI < 25, Overweight = 25 <= BMI < 30, Obesity = BMI > 30; Smoking status (never, former, current smoker); Alcohol intake at baseline: < 1 g/day is considered non-drinker; PA Index: physical activity, according to the Cambridge physical activity Index; mrMDS: modified Mediterranean dietary score. RII in tertiles: RII = 1 (High Socioeconomic Position (SEP)), RII = 2 (Medium SEP), RII = 3 (Low SEP).

## RESULTS

**Figure 1.**
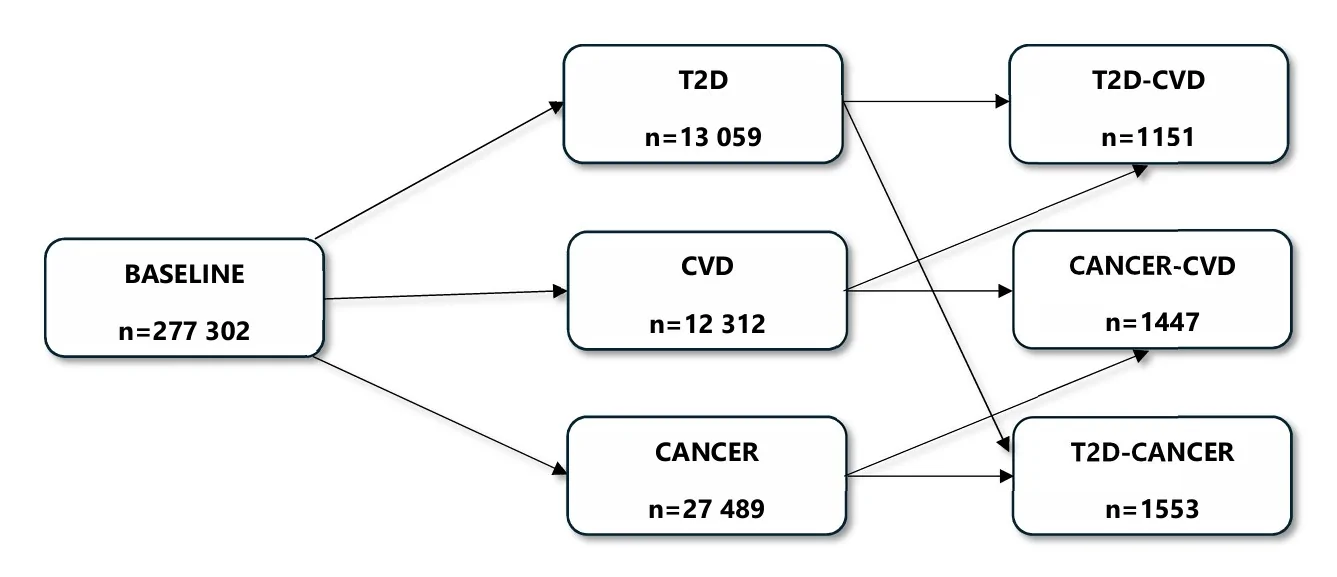
**Number of events for each state from disease-free state to first diagnosis and multimorbidity**. T2D: Type 2 Diabetes; CVD: Cardiovascular Disease.

The sample analysed wad composed by 168 270 women (60.68%) and 109 032 men (39.28%). The median age was 52.08 years in women (Interquartile range (IQR) = (45.12 - 58.62)) and 52.75 years in men (IQR = (46.71 - 59.00)). In total, 13 059 (4.71%) cases of T2D, 12 312 (4.44%) cases of CVD and 27 489 (9.91%) cases of cancer were registered as first diagnosis. Moreover, 4 151 (1.50%) developed multimorbidity during the follow up (Fig. 1). The frequencies and proportions for the variables of interest are shown in Table 1. The preliminary results from the MCA and subsequent clustering brought to the identification of 6 multimorbidity clusters for women and 5 clusters for men (Supplementary Fig. 13 - 23). In particular, melanoma tended to cluster into a single group in the women’s sample, separated by any other cancer diagnosis. The same behaviour was not found in men, even though the proportion of melanoma cases was similar across sexes. Stroke tended to occur together with breast cancer in women and with prostate cancer in men. In contrast, in clusters where T2D was the most prevalent condition, no women with breast cancer nor men with prostate cancer were found. In regards to the distribution of RII within each cluster, in the women’s melanoma cluster, women tended to have a higher SEP than the entire women sample (Supplementary Table 7), while men in the T2D cluster had lower SEP level compared to the male sample as a whole (Supplementary Table 8). For a detailed descriptive analysis of multimorbidity cases, see the supplementary material.

Upon examining the entire sample, the multistate model revealed significant differences in risk for all 9 transitions. Regarding lifestyle factors (BMI, smoking status, mrMDS, physical activity and alcohol intake), the results of the main model are shown in Table 2 (for transitions from disease-free to first diagnosis) and Table 3 (for transitions to multimorbidity). Overall, the majority of lifestyle factors were risk factors for all the transitions from disease-free state. For the transitions to multimorbidity, BMI and smoking habits remained significant risk factors, while mrMDS and physical activity were not. Moreover, for alcohol intake, no association with multimorbidity was found and for transitions from disease-free state to first diagnosis it even seemed to act as a protective factor.

**Table 2:** Hazard ratios (HR) for lifestyle variables across the transitions from disease free to a first diagnosis, total sample.

		DISEASE-FREE → T2D	DISEASE-FREE → CVD	DISEASE-FREE → CANCER
		HR (95% CI)	HR (95% CI)	HR (95% CI)
**BMI**	Normal	1 (Ref.)	1 (Ref.)	1 (Ref.)
	Overweight	3.22 (3.05-3.41)	1.40 (1.34-1.47)	1.08 (1.05-1.11)
	Obesity	9.27 (8.75-9.82)	1.65 (1.56-1.75)	1.16 (1.11-1.21)
**Smoking**	Non-smoker	1 (Ref.)	1 (Ref.)	1 (Ref.)
	Former smoker	1.13 (1.08-1.19)	1.18 (1.12-1.24)	1.14 (1.00-1.17)
	Current smoker	1.26 (1.20-1.32)	1.86 (1.77-1.95)	1.29 (1.25-1.33)
**mrMDS**	High	1 (Ref.)	1 (Ref.)	1 (Ref.)
	Medium	1.03 (0.98-1.09)	1.13 (1.06-1.21)	1.02 (0.98-1.06)
	Low	1.16 (1.08-1.23)	1.18 (1.00-1.27)	1.07 (1.03-1.23)
**Pa Index**	Active	1 (Ref.)	1 (Ref.)	1 (Ref.)
	Moderately active	1.09 (1.03 -1.16)	1.10 (1.04-1.17)	1.08 (1.04-1.13)
	Moderately inactive	1.23 (1.17-1.30)	1.23 (1.17-1.30)	1.16 (1.11-1.20)
	Inactive	1.48 (1.40-1.57)	1.60 (1.51-1.70)	1.35 (1.29-1.41)
**Alcohol**	Non-drinker (<1 g/day)	1 (Ref.)	1 (Ref.)	1 (Ref.)
	Men 1-24 g/day; women 1-12 g/day	0.79 (0.74-0.84)	0.77 (0.72-0.82)	0.93 (0.89-0.97)
	Men >= 24 g/day; women >= 12 g/day	0.82 (0.77-0.87)	0.84 (0.78-0.89)	0.93 (0.89-0.97)

T2D: Type 2 Diabetes; CVD: Cardiovascular Disease; BMI, Body Mass Index: Normal = BMI < 25, Overweight = 25 <= BMI < 30, Obesity = BMI > 30; Smoking status (never, former, current smoker); Alcohol intake at baseline: < 1 g/day is considered non-drinker; PA Index: physical activity, according to the Cambridge physical activity Index; mrMDS: modified Mediterranean dietary score.

**Table 3:** Hazard ratios (HRs) for lifestyle variables across the transitions from first diagnosis to multimorbidity, total sample.

		T2D → CVD	T2D → CANCER	CVD → T2D	CVD → CANCER	CANCER → CVD	CANCER → T2D
		HR (95% CI)	HR (95% CI)	HR (95% CI)	HR (95% CI)	HR (95% CI)	HR (95% CI)
**BMI**	Normal	1 (Ref.)	1 (Ref.)	1 (Ref.)	1 (Ref.)	1 (Ref.)	1 (Ref.)
	Overweight	1.21 (0.93-1.58)	1.00 (0.82-1.22)	2.34 (1.82-3.01)	0.95 (0.81-1.11)	1.06 (0.89-1.27)	3.15 (2.48-4.00)
	Obesity	1.13 (0.86-1.49)	0.92 (0.76-1.13)	5.25 (4.02-6.85)	1.23 (1.00-1.51)	1.48 (1.17-1.87)	7.62 (5.93-9.79)
**Smoking**	Non-smoker	1 (Ref.)	1 (Ref.)	1 (Ref.)	1 (Ref.)	1 (Ref.)	1 (Ref.)
	Former smoker	1.03 (0.83-1.29)	1.09 (0.92-1.28)	1.20 (0.95-1.52)	0.98 (0.81-1.18)	1.07 (0.88-1.30)	0.99 (0.80-1.21)
	Current smoker	1.62 (1.32-2.00)	1.32 (1.12-1.56)	1.37 (1.10-1.71)	1.20 (1.00-1.42)	1.43 (1.17-1.74)	1.23 (1.00-1.51)
**mrMDS**	High	1 (Ref.)	1 (Ref.)	1 (Ref.)	1 (Ref.)	1 (Ref.)	1 (Ref.)
	Medium	0.94 (0.72-1.23)	1.08 (0.87-1.35)	1.08 (0.81-1.46)	0.86 (0.68-1.09)	0.95 (0.73-1.24)	0.88 (0.68-1.13)
	Low	1.11 (0.81-1.51)	1.18 (0.93-1.51)	1.09 (0.78-1.52)	0.92 (0.71-1.86)	1.24 (0.93-1.66)	1.11 (0.84-1.48)
**Pa Index**	Active	1 (Ref.)	1 (Ref.)	1 (Ref.)	1 (Ref.)	1 (Ref.)	1 (Ref.)
	Moderately active	1.05 (0.80-1.37)	0.98 (0.80-1.20)	0.81 (0.63-1.04)	1.09 (0.88-1.36)	0.98 (0.77-1.27)	0.87 (0.67-1.11)
	Moderately inactive	1.12 (0.88-1.43)	1.12 (0.94-1.35)	0.94 (0.74-1.18)	1.03 (0.84-1.26)	1.05 (0.83-1.32)	0.91 (0.73-1.16)
	Inactive	1.40 (1.08-1.80)	1.15 (0.95-1.41)	0.98 (0.76-1.26)	1.33 (1.08-1.65)	1.21 (0.94-1.55)	0.94 (0.73-1.22)
**Alcohol**	Non-drinker (<1 g/day)	1 (Ref.)	1 (Ref.)	1 (Ref.)	1 (Ref.)	1 (Ref.)	1 (Ref.)
	Men 1-24 g/day; women 1-12 g/day	1.05 (0.78-1.39)	1.02 (0.80-1.29)	0.99 (0.74-1.33)	0.90 (0.72-1.14)	0.80 (0.61-1.05)	0.76 (0.58-1.00)
	Men >= 24 g/day; women >= 12 g/day	1.08 (0.82-1.43)	1.10 (0.88-1.38)	0.99 (0.74-1.32)	0.83 (0.66-1.03)	0.93 (0.72-1.20)	0.82 (0.63-1.07)

T2D: Type 2 Diabetes; CVD: Cardiovascular Disease; BMI, Body Mass Index: Normal = BMI < 25, Overweight = 25 <= BMI < 30, Obesity = BMI > 30; Smoking status (never, former, current smoker); Alcohol intake at baseline: < 1 g/day is considered non-drinker; PA Index: physical activity, according to the Cambridge physical activity Index; mrMDS: modified Mediterranean dietary score.

Table 4 and Table 5 focus on the results of SEP, for the whole sample and according to sex. Having a low or medium SEP (RII = 3 and RII = 2) compared to having a high SEP (RII = 1) was associated with a higher risk of transitioning to a T2D diagnosis by 26% and 15%, respectively (hazard ratio (HR): 1.26, CI: 1.20-1.32; HR: 1.15, CI: 1.10-1.21). Similarly, in the transition to a CVD the risk for the subjects with the lowest RII level was higher (HR: 1.31, CI: 1.25-1.38) compared to the subjects in the first RII tertile. The risk for the transition to cancer instead was not statistically significant. Similar results have been found in the secondary models, with a significant association between SEP and the risk of transitioning from disease-free state to CVD for men (HR: 1.26, CI: 1.19-1.35) and women (HR: 1.35, CI: 1.25-1.45), considering the lowest RII level.

Transitioning from disease-free state to cancer, we found that a lower SEP level was a protective factor for women in both RII = 2 and RII = 3 levels compared to High SEP, while it was a significant risk factor for men, but only for men in the lowest RII tertile.

**Table 4:** Hazard ratios (HR) for the Socioeconomic Position (SEP) across the transitions from disease free to a first diagnosis, total sample and by sex.

		DISEASE-FREE → T2D	DISEASE-FREE → CVD	DISEASE-FREE → CANCER
		HR (95% CI)	HR (95% CI)	HR (95% CI)
**Total**	RII = 1	1 (Ref.)	1 (Ref.)	1 (Ref.)
	RII = 2	1.15 (1.10-1.21)	1.06 (1.01-1.12)	0.96 (0.92-0.99)
	RII = 3	1.26 (1.20-1.32)	1.31 (1.25-1.38)	1.01 (0.98-1.05)
**Women**	RII = 1	1 (Ref.)	1 (Ref.)	1 (Ref.)
	RII = 2	1.13 (1.05-1.21)	1.03 (0.95-1.16)	0.92 (0.88-0.96)
	RII = 3	1.23 (1.14-1.32)	1.35 (1.25-1.45)	0.94 (0.90-0.98)
**Men**	RII = 1	1 (Ref.)	1 (Ref.)	1 (Ref.)
	RII = 2	1.17 (1.10-1.26)	1.08 (1.01-1.15)	1.00 (0.95-1.05)
	RII = 3	1.27 (1.19-1.36)	1.26 (1.19-1.35)	1.11 (1.06-1.17)

T2D: Type 2 Diabetes; CVD: Cardiovascular Disease; Relative Index of Inequality (RII) in tertiles: RII = 1 (High Socioeconomic Position (SEP)), RII = 2 (Medium SEP), RII = 3 (Low SEP).

**Figure 2.**
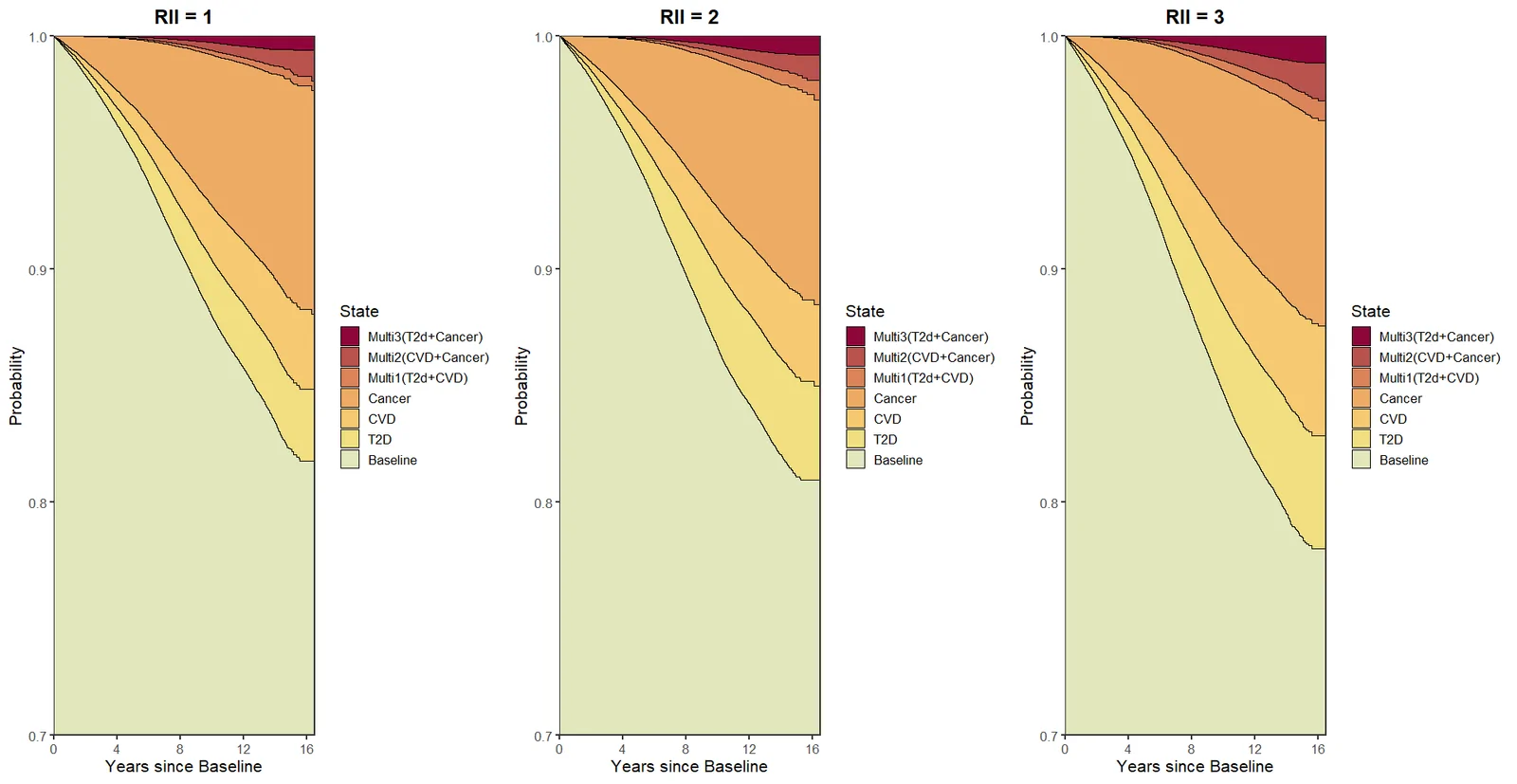
**Stacked transition probabilities by Relative Index of Inequality (RII) tertiles**. Each figure shows the probability to belong to a particular state during the follow-up time, having a certain level of socioeconomic position (SEP). T2D: Type 2 Diabetes; CVD: Cardiovascular Disease; RII = 1 (High SEP), RII = 2 (Medium SEP), RII = 3 (Low SEP).

Comparing all the transitions from the first diagnosis to multimorbidity, the HRs for the lowest RII tertile in the transitions to CVD were as follows: HR T2D to CVD: 1.20, CI: 0.97-1.49; HR cancer to CVD: 1.23, CI: 1.02-1.50. Both the transitions to T2D showed a positive association, with HRs of 1.35 (CI: 1.07-1.71) and 1.37 (CI: 1.10-1.69) for CVD and cancer patients respectively. On the contrary, in the transition from CVD to cancer (HR: 0.96, CI: 0.80-1.14) and from T2D to cancer (HR: 1.08, CI: 0.92-1.28) the results were not significant. Moreover, in the secondary models (after splitting by sex), the majority of results were not significant, although this could be due to a reduced sample size. In particular, the only significant transitions in the women sample were the transition from cancer to T2D (HR = 1.45; CI: 1.07-1.96) and from T2D to CVD (HR = 1.56; CI; 1.06-2.29). In Figure 2, the transition probabilities calculated for the different tertiles of the RII variable are shown. The transition probabilities along the whole follow up were higher for the subjects in the lower tertiles of the RII distribution.

**Table 5:** Hazard ratios (HR) for the Socioeconomic Position (SEP) across the transitions from first diagnosis to multimorbidity, total sample and by sex.

		T2D → CVD	T2D → CANCER	CVD → T2D	CVD → CANCER	CANCER → CVD	CANCER → T2D
		HR (95% CI)	HR (95% CI)	HR (95% CI)	HR (95% CI)	HR (95% CI)	HR (95% CI)
**Total**	RII = 1	1 (Ref.)	1 (Ref.)	1 (Ref.)	1 (Ref.)	1 (Ref.)	1 (Ref.)
	RII = 2	1.11 (0.88-1.40)	0.91 (0.76-1.08)	1.55 (1.22-1.98)	0.91 (0.76-1.10)	0.88 (0.71-1.10)	1.27 (1.01-1.59)
	RII = 3	1.20 (0.97-1.49)	1.08 (0.92-1.28)	1.35 (1.07-1.71)	0.96 (0.80-1.14)	1.23 (1.02-1.50)	1.37 (1.10-1.69)
**Women**	RII = 1	1 (Ref.)	1 (Ref.)	1 (Ref.)	1 (Ref.)	1 (Ref.)	1 (Ref.)
	RII = 2	1.42 (0.94-2.13)	1.07 (0.80-1.43)	1.39 (0.90-2.16)	1.15 (0.84-1.59)	0.81 (0.58-1.13)	1.20 (0.87-1.66)
	RII = 3	1.56 (1.06-2.29)	1.08 (0.82-1.43)	1.36 (0.91-2.03)	1.07 (0.79-1.44)	1.22 (0.90-1.65)	1.45 (1.07-1.96)
**Men**	RII = 1	1 (Ref.)	1 (Ref.)	1 (Ref.)	1 (Ref.)	1 (Ref.)	1 (Ref.)
	RII = 2	0.99 (0.75-1.30)	0.81 (0.65-1.02)	1.62 (1.20-1.17)	0.80 (0.64-1.02)	0.93 (0.70-1.25)	1.36 (0.99-1.86)
	RII = 3	1.06 (0.82-1.38)	1.08 (0.88-1.33)	1.35 (1.00-1.80)	0.91 (0.73-1.12)	1.23 (0.95-1.58)	1.31 (0.97-1.74)

T2D: Type 2 Diabetes; CVD: Cardiovascular Disease; Relative Index of Inequality (RII) in tertiles: RII = 1 (High Socioeconomic Position (SEP)), RII = 2 (Medium SEP), RII = 3 (Low SEP).

## DISCUSSION

The results from this cohort study suggested that SEP was an independent predictor of the transition from the disease-free condition to one of the 3 conditions studied and subsequently to multimorbidity. Moreover, lifestyle factors contributed to the risk of developing NCDs and multimorbidity, possibly acting as individual contributors.

Regarding multimorbidity, for both the transitions from T2D and CVD to cancer, the association with SEP was not present while in the transition from cancer to T2D, it remained significant with strong evidence for both men and women. As for the cancer to CVD transition, we found a borderline significant association for the lowest RII tertile, when considering the whole sample.

The role of SEP in relation to risk of cancer in T2D patients is in line with other findings, where SEP has found to play a small role for people with T2D in terms of their risk of cancer [35] and in affecting the risk of cancer mortality in T2D patients [36]. On the other hand, the inequalities observed in the transition from cancer to T2D are not surprising: it is well known that cancer and T2D share many common risk factors [37], that can in turn increase the inequalities associated with a lower SEP in cancer patients, who are in general at higher risk of diabetes. It is possible that mediator factors are still important in the risk of developing T2D after a cancer diagnosis, whereas in the opposite direction different mechanisms, unrelated to SEP, become more significant. Furthermore, although evidence that inequalities across SEP groups are still in place in secondary prevention, even in countries with universal health coverage [38], it is reasonable to suppose that general SEP inequalities would shrink compared to those present the general population. On the other hand, it has been suggested that certain treatments, e.g. certain insulin therapies, especially high doses, may be associated with an increased risk of certain cancers [39, 40].

In both directions of the transition from cancer to CVD and from CVD to cancer, we could not find any association between SEP and multimorbidity, except for a borderline significant estimate in the whole sample when comparing low SEP to high SEP in the cancer to CVD transition. This result could partly be due to the fact that the death incidence rates were much higher for cancer and CVD patients than for T2D patients. Thus, the competing risk of mortality was comparatively larger among individuals with cancer and CVD than among those with T2D, which may have attenuated the estimates.

In T2D patients, SEP was not a significant predictor of CVD, with only a borderline association observed in women within the lowest RII tertile. This may be because, in subjects with T2D, social inequalities are of secondary importance compared to mechanisms unrelated to socioeconomic factors, such as genetic and metabolic aspects [41, 42]. However, among women, several factors, such as medication adherence and side effects, CVDs management and psychological aspects, pose different levels of CVD risk compared to men. These factors could potentially mediate the association between SEP and CVDs in women with T2D, explaining the positive gradient observed in the women’ sample [43]. On the contrary, in CVD patients, a strong association between SEP and the transition to T2D was found for the entire sample and for the men sample. The estimates reflect the results of the transition from disease-free status to T2D, as well as previous findings [20]. The observed sex differences may mirror the fact that T2D diabetes is more prevalent in men than in women worldwide and that men are typically diagnosed at a younger age and with a lower BMI [44].

The social inequalities in the risk of transitioning from a disease-free state to T2D were similar for men and women and confirmed previous findings [20]. Moreover, the estimates showed a significant association for the whole sample between SEP and risk of a CVD diagnosis. After considering women and men separately, the association was slightly stronger for the latter group, as the results were significant for both the medium and lower RII. Usually, men have a higher incidence of CVD [45] and develop CVD, in particular coronary heart disease, at a younger age than women [46]. The differences are to be sought both in genetics and environmental causes [45, 47], with some shared risk factors like smoking, hypertension and overweight found to be more common in men [47]. However, the gender gap for some CVDs like stroke becomes minor in older age [46]. In addition, CVD risk factors like smoking and hypertension are more harmful in women. In particular, hypertension is common in post-menopausal women and significantly increases the risk of left ventricular hypertrophy, leading to a higher incidence of heart failure and stroke. Moreover, smoking appears to be more harmful in women due to faster nicotine metabolism and the effects of burning products [48]. The literature on gender differences in socioeconomic inequalities in CVDs is inconsistent, with a meta-analysis finding a stronger gradient for women [49].

Overall, estimates for the transition from disease-free to cancer diagnosis in the whole sample were not significant, which reflects the positive gradient between SEP and risk of cancer in women. A lower risk of cancer in women with low SEP has been found before in the EPIC cohort [50] and is at least partially explained by reproductive factors: women with higher SEP tend to have less children and later in life [50]. Also, the use of hormone therapy is reported to be more common in women with high SEP [51]. Both these factors are risk factors for post-menopausal breast cancer [50, 51], the most common cancer diagnosed in European women (*Cancer in Europe: 5 things the data tells us.* European Commission; https://joint-research-centre.ec.europa.eu/jrc-news-and-updates/cancer-europe-5-things-data-tells-us-2022-01-13_en. Accessed 13 Jun 2023). Moreover, we found that in the melanoma cluster, women tended to have a higher SEP compared to the overall female sample. This is consistent with findings from a systematic review [52]. While a lower SEP has been associated with later-stage disease at time of diagnosis and greater mortality, the increased risk of this type of cancer in individuals with higher SEP cannot be entirely attributed to greater vigilance and awareness among this group. Instead, it may also be explained by lifestyle factors and their interaction with SEP, including occupation, occupational exposure, recreational sun exposure and tanning.

### Strength and limitation

The present study has several strengths: individual level data has been used, from a very large European cohort originally designed for cancer studies, with validated diagnoses of T2D and CVDs and information on pre-diagnostic exposure to several lifestyle factors. Moreover, the associations were modelled in a multi-state framework to consider the possible sequences of the incident chronic conditions, avoiding restricting the definition of multimorbidity as the sum of different and often equally important diseases. Finally, the prospective design of the study allows for examining temporality between SEP and multimorbidity.

However, several limitations have to be taken into consideration when evaluating the results: first, information on lifestyle was only collected at baseline, and the participants could have changed their lifestyle habits, especially after the first diagnosis, which we were not able to capture. However, it has previously been shown that in absence of interventions, the vast majority of individuals do not make lifestyle changes after the diagnosis of the first NCD [53]. A second limitation concerns the lack of information on treatment after the first NCD diagnosis, which could have modified the risk of subsequent multimorbidity [54, 55]. For example, it is well known that CVD risk increases with cancer therapy [56]. If the treatment should be considered independent of SEP is debatable, even in countries where healthcare coverage is granted for anybody. A third aspect is the use of RII as the only proxy for SEP. While the strengths of this choice have been already discussed and other metrics like occupation and income are strongly correlated to each other and to the educational level [57], they do not overlap completely. Therefore, it is possible that we were not able to capture the whole impact of the socioeconomic determinants by RII. Fourth, although the sampling of participants into EPIC was conducted from the general population, the response rate seemed to vary according to education level, with lower educated people having a lower response rate. Usually, the response rate is lower for people with low SEP in lifestyle surveys [58] and that can bias the results towards the null. Fifth, an important part of the study is based on self-reported conditions and habits, potentially introducing some bias linked with education [59]. However, all the self-reported cancer cases were verified through medical records and validation studies have been conducted for T2D, myocardial infarction (MI) and stroke cases. Finally, other diseases such as dementia, chronic obstructive pulmonary disease (COPD) and other chronic respiratory diseases were not considered in the definition of multimorbidity, due to lack of information. However, despite the large burden of those diseases, CVDs, cancer and T2D are among the top four conditions responsible for the highest number of annual global deaths (*Non communicable diseases.* WHO; www.who.int/news-room/fact-sheets/detail/noncommunicable-diseases. Accessed 2 Jun 2023).

## Conclusion

The findings of this study suggest that SEP remains a driver of social inequalities in health even after the first NCD diagnosis. This implies that even in a subset of the population already diagnosed with one NCD, which is therefore supposed to be followed more closely by the healthcare system, notable differences in terms of multimorbidity risk endure across socioeconomic strata. These results support health policies implementation to bridge the gap between different socioeconomic groups, by implementing specific measures targeted at the most vulnerable layers of the population.

## Supplementary Materials

The Supplementary data can be found online at: www.aginganddisease.org/EN/10.14336/AD.2024.1166.
