# Supplementary material for: A longitudinal Analysis of the Association between Socioeconomic Position and Multimorbidity in the European Prospective Investigation into Cancer and Nutrition Study
Source: Aging Dis. 2024 Dec 3;16(6):3625–37. doi: 10.14336/AD.2024.1166 (PMC12539534; doi:10.14336/AD.2024.1166)
Supplement: Supplementary file 1 — The Supplementary data can be found online at: www.aginganddisease.org/EN/10.14336/AD.2024.1166. [file AD-16-6-3625-s.pdf]

SUPPLEMENTARY DATA

A longitudinal Analysis of the Association between Socioeconomic Position and Multimorbidity in the European Prospective Investigation into Cancer and Nutrition Study

Luca Manfredi, Barbara Sodano, Chiara Raganato, Federica Buscema, Lorenzo Milani, Alberto Catalano, Heinz Freisling, Pietro Ferrari, Alem Abraha, Rudolf Kaaks, Verena Katzke, Salvatore Panico, Christian Skødt Antoniussen, Christina C. Dahm, Sandar Tin Tin, Roel Vermeulen, Ilonca Vaartjes, Anne Tjønneland, Anja Olsen, Sandra Colorado-Yohar, Sara Grioni, Marc J Gunter, Matthias B. Schulze, Reynalda Cordova, Maria-Jose Sánchez, Catalina Bonet Bonet, Rosario Tumino, Olatz Mokoroa, Giovanna Masala, Marcela Guevara Eslava, Monique Verschuren, Carlotta Sacerdote, Fulvio Ricceri

© 2024. Manfredi L. et al. Published online at http://www.aginganddisease.org/EN/10.14336/AD.2024.1166

SUPPLEMENTARY DATA

Supplementary Methods

In the descriptive and cluster analysis of multimorbidity (MM), stroke and coronary heart disease (CHD) cases have been left separated. In this context, MM defined as the presence of more than 2 diseases has also been considered, allowing for the separation of subjects with 3 or 4 diseases. Moreover, the overall number of persons with MM is slightly higher in this framework compared to the main analysis, since for the total sample only the first event between CHD and stroke was taken into consideration. For conducting the analyses, an additional dichotomous variable indicating the presence/absence of MM was constructed. The presence of MM was characterized by having two or more of the following conditions: cancer, type 2 diabetes (T2D), CHD, and stroke. The Relative index of inequality (RII) variable was divided into tertiles as in the main analysis.

By exploiting original morphological data, stage, site and diagnosis date, 25 types or groups of cancer have been codified: leukemia, lymphoma, melanoma, oral, cervix, colorectum, duodenum, brain, esophagus, liver, larynx, breast, ovarian, uterine, pancreas, lung, prostate, kidney, stomach, thyroid, soft tissue, bladder, other digestive, other female genitalia, other cancers. Cancer types were further grouped for the analysis, by shared risk factors and body system. In the women's dataset, this grouping led to the identification of 13 groups of tumors (mouth and larynx, cervix, colorectal, body of the uterus, liver and pancreas, leukemia and lymphoma, breast, melanoma, ovary and other genital organs, lung, digestive tract, bladder and kidneys, other cancers), whereas 10 were identified for the men's dataset (mouth and larynx, colorectal, liver and pancreas, leukemia and lymphoma, melanoma, lung, digestive tract, bladder and kidneys, prostate, other cancers).

As the first step for the cluster analysis of MM, a dimensionality reduction with Multiple Correspondence Analysis (MCA) was performed. MCA generalizes principal component analysis (PCA), looking for potential relationships between multiple categorical variables. Similar to PCA, MCA dimensions are orthogonal to each other and are selected and sorted in order to explain as much of the variance as possible. By applying the Singular Value Decomposition to the standardized residuals matrix, MCA reduces the number of dimensions in a given dataset and obtains the principal components (factors) and the factor scores. The data dimension reduction allows for the visual representation of both variables and observations on a two-dimensional plane [1]. Afterwards, hierarchical clustering on principal components has been performed using the FactoMineR package [2]. MCA is utilized as a pre-processing method to convert categorical data into continuous variables and also to perform a dimensionality reduction in order to avoid the distance concentration effect that can reduce the clustering effectiveness. The analysis was run separately for men and women. For the selection of the principal components, a threshold of 45% cumulative inertia was chosen. Next, a hierarchical clustering algorithm was applied. Euclidean distance was used as the measure of dissimilarity, and average linkage was used as the measure of linkage. Finally, a chi-squared test was performed to assess the association between SEP levels and different clusters.

Supplementary Results

The dataset of women with MM consisted of 2 395 individuals; among these, 1 857 (77.54%) had cancer, 1 422 (59.37%) had T2D, 935 (39.04%) had CHD and 720 (30.06%) had a stroke. The dataset of men with MM consisted of 3 859 individuals; among these, 2 899 (75.12%) had cancer, 2 216 (57.42%) had T2D, 1 787 (46.31%) had CHD, and 1 147 (29.72%) had a stroke. Table S1 and S2 present the distribution of the RII divided into tertiles, separately for each tumor or tumor group. The observed frequencies, both in the female and male datasets, suggest generally lower levels of education among individuals affected by cancer.

MCA allowed the identification of 6 factors for the women's group and 5 for the men's group, respectively. The number of factors was selected based on the cumulative inertia explained by the factors themselves. In tables S5 and S6, the results are presented, showing the proportion of inertia from the original dataset explained by each factor and the cumulative inertia, separately for women and men. In both cases, the number of factors was chosen based on the cumulative percentage of explained inertia: in the first case, the first 6 factors accounted for approximately 45% of the variability of the original dataset, while for the men's dataset, the first 5 factors accounted for approximately 47%. The meaning of each factor was attributed based on the contribution of individual modalities to the factor itself: figure S2 – S12 show bar charts indicating the modalities that contribute most to defining each of the factors. The dashed horizontal line indicates the expected value in the hypothetical case where the contribution is uniform across all modalities.

© 2024. Manfredi L. et al. Published online at http://www.aginganddisease.org/EN/10.14336/AD.2024.1166

Therefore, in general, for a given dimension, any modality that exhibits a contribution above the reference line could be considered important in defining the factor itself.

The analysis identified 6 clusters through hierarchical clustering: the first cluster consists of 454 women, the second of 850, the third of 232, the fourth of 157, the fifth of 527, and the sixth of 175. The first cluster can be identified as the breast cancer cluster: it comprises 454 women with breast cancer, of whom 305 (67.2%) also have T2D, 137 (30.2%) also have CHD, and 12 (2.6%) have all three conditions listed above (Figure S13). The second cluster, consisting of 850 women, could be considered the T2D and CHD group: about 70% of the women in the cluster have T2D, while about 50% have CHD. In this cluster, 355 women (41.8%) have T2D and cancer, 237 (27.9%) have CHD and T2D, 167 (19.6%) have both CHD and cancer, 47 women (5.5%) have stroke and cancer, and 44 (5.2%) have three of the four chronic conditions considered. The cancers (or groups of cancers) clustered in this group are as follows: lung, body of the uterus, leukemia and lymphoma, liver and pancreas, bladder and kidneys, ovary and other female genital organs, cervix, digestive tract, mouth, and larynx. As described in Figure S14, 252 women do not have cancer, while most women in the cluster have lung cancer (166), followed by uterine body cancer (90), and leukemia and lymphoma (89). The third cluster can be identified as the colorectal cancer group: as shown in Figure S15, it comprises 232 women with this cancer. Of these, 110 (47.7%) also have T2D, 64 (27.6%) also have CHD, 38 (16.4%) also suffer from stroke, and 20 women (8.6%) have three of the four chronic conditions considered. The fourth cluster (Figure S16), consisting of 157 women, is the other cancers group: of these, 73 (46.5%) also have T2D, 39 (24.8%) also have CHD, 34 (21.7%) have both stroke and other cancers, while 11 women (7%) have three of the four conditions considered. The other cancer groups, includes soft tissue, brain, thyroid, and other rare cancers coded other. The fifth cluster could be interpreted as the group of women with CVDs. As shown in Figure S17, it consists of 527 women, all of whom have at least one condition between stroke and CHD. Specifically, 360 women (68.3%) have both stroke and cancer, 127 (24.1%) have both stroke and CHD, 37 women (7%) have three chronic conditions, and 3 women (0.6%) have four. The cancers grouped in the fifth cluster are as follows: breast, leukemia, lymphoma, body of the uterus, liver, pancreas, ovary, other female genital organs, bladder, kidneys, digestive tract, cervix, mouth, and larynx. Among the cancers, breast cancer is the most frequent in the cluster, accounting for about half of the total cancers, and all women with breast cancer also suffer from stroke. Finally, the sixth group (Figure S18) identified is the melanoma cluster, consisting of 175 women. All women in the cluster have melanoma, of whom 68 (38.9%) also have T2D, 60 (34.3%) also have CHD, 34 (19.4%) women suffer from both melanoma and stroke, 12 (6.9%) have three chronic conditions, and 1 woman (0.6%) has four.

The analysis on the male sample, using hierarchical clustering, identified 5 distinct clusters: the first cluster consists of 1354 men, the second of 293, the third of 693, the fourth of 376, and the fifth of 1143 men. The first resulting group, composed of 1354 men with MM, can be identified as the CVDs cluster, as all individuals in the cluster have at least one condition between stroke and CHD. As shown in Figure S19, 625 men (46.2%) report stroke and cancer, 356 men (26.3%) have both CHD and cancer, 213 (15.7%) have both CHD and stroke, 155 men (11.4%) have three concomitant chronic conditions, while 5 (0.4%) have four. In this cluster, the cancers grouped are as follows: prostate, melanoma, lung, other cancers, leukemia, lymphoma, digestive tract, mouth, larynx, liver, and pancreas. The most frequent cancer in the cluster is prostate cancer, followed by melanoma and lung cancer. The second cluster can be summarized as the bladder and kidney cancer group, comprising 293 men with one of these two cancers. As shown in Figure S20, 109 men (37.2%) also have T2D, 102 (34.8%) have both bladder or kidney cancer and CHD, 53 men (18.1%) also have stroke, and 29 men (9.9%) have three chronic conditions. The third cluster, composed of 693 individuals, is the prostate cancer cluster. As shown in Figure S21, all 693 men in the group suffer from prostate cancer, of whom 53.7% (372) also have T2D, 40.8% (283) also have CHD, while 38 men (5.5%) have three concomitant chronic conditions. No individuals in this cluster suffer from stroke. The fourth cluster is the colorectal cancer group, composed of 376 men with this condition. As shown in Figure S22, 48.9% (184) of these men also have T2D, 15.7% (59) also have stroke, 27.4% (103) have both CHD and colorectal cancer, 29 men (7.7%) have three chronic conditions, while one man (0.3%) has four. Finally, the fifth cluster, composed of 1143 men, can be considered the T2D group, as more than 90% of the men in this cluster have this chronic condition. As shown in Figure S23, 588 men (51.4%) have both T2D and cancer, 464 (40.6%) have both T2D and CHD, 24 men (2.1%) have both CHD and cancer, 66 men (5.8%) have three chronic conditions, while one man (0.1%) has four. The cancers grouped in this cluster are as follows: liver and pancreas (in higher percentage), melanoma, lung, other cancers, leukemia, lymphoma, digestive tract, mouth, and larynx.

Following the observation of the constructed clusters, which allowed the assessment of which individuals with different pathologies were more similar to each other, the distribution of the RII within the defined clusters was evaluated. Table S7 shows the distribution of the RII divided into tertiles separately for each cluster in the women's

dataset. The chi-squared test result was borderline significant (p-value = 0.0426). As highlighted in the table, 23.88% of the entire women's dataset had an RII level of 1 (high level), 31.77% had a medium level (RII = 2), and 44.34% had a low RII level (RII = 3). Comparing the distribution of the RII in each cluster with the distribution of the total dataset, it was observed that the sixth cluster, the group containing women with melanoma, was more educated compared to the total group of women with MM (31.43% vs. 23.88% for RII=1; 40.57% vs. 44.34% for RII=3). The fourth cluster, the group of women with other cancers (soft tissues, brain, thyroid, and other cancers), included less educated women compared to the general group: 17.2% of the cluster had RII=1 (high level) compared to 23.88% of the general group, while 50.96% had RII=3 (low level) compared to 44.34% of the total group of women with MM. The RII distribution in the remaining clusters aligned with that of the total dataset.

Table S8 shows the same distribution for the men's dataset. In the entire male dataset, 26.12% had an RII level of 1 (high level), 30.29% had a medium level (RII = 2), and 43.59% had a low RII level (RII = 3). The total results were in line with the previously shown women's dataset. Unlike the clusters of MM in the women's dataset, there were no significant situations where specific pathology groups present significant differences in the RII distribution, except for the fifth cluster, the T2D cluster, where a lower percentage of men with RII = 1 (high level) suggests a generally lower level of education for this group compared to other groups and the total group of men with MM. Each cluster aligned with the distribution of the total dataset of men with concurrent chronic conditions. The chi-squared test to evaluate the relationship between RII and constructed clusters resulted in a p-value of 0.0023.

Supplementary References

[1] Abdi H, Valentin D Multiple Correspondence Analysis. Mult. Corresp. Anal.
[2] Lê S, Josse J, Husson F (2008). FactoMineR: An R Package for Multivariate Analysis. J Stat Softw, 25:1–18.

Supplementary Table 1. Distribution of the 13 cancer groups in women by the Relative Index of Inequality (RII) tertiles

**RII**	**1**	**2**	**3**	**Total**
**Cancer groups**	**N (%)**	**N (%)**	**N (%)**	**N (%)**
Mouth and Larynx	3 (11.11%)	10 (37.04%)	14 (51.85%)	27 (100.00%)
Cervix	9 (24.32%)	13 (35.14%)	15 (40.54%)	37 (100.00%)
Colorectal	62 (26.72%)	73 (31.47%)	97 (41.81%)	232 (100.00%)
Body of the Uterus	24 (20.87%)	48 (41.74%)	43 (37.39%)	115 (100.00%)
Liver and Pancreas	28 (29.79%)	32 (34.04%)	34 (36.17%)	94 (100.00%)
Leukemia and Lymphoma	27 (23.68%)	36 (31.58%)	51 (44.74%)	114 (100.00%)
Breast	151 (26.63%)	184 (32.45%)	232 (40.92%)	567 (100.00%)
Melanoma	55 (31.43%)	49 (28.00%)	71 (40.57%)	175 (100.00%)
Ovary and other genital organs	14 (22.95%)	23 (37.70%)	24 (39.34%)	61 (100.00%)
Lung	34 (20.48%)	48 (28.92%)	84 (50.60%)	166 (100.00%)
Digestive Tract	9 (23.68%)	14 (36.84%)	15 (39.47%)	38 (100.00%)
Bladder and Kidneys	16 (21.62%)	20 (27.03%)	38 (51.35%)	74 (100.00%)
Other Cancers	27 (17.20%)	50 (31.85%)	80 (50.96%)	157 (100.00%)

Digestive tract includes stomach, esophagus, duodenum, and other digestive cancers, while other cancers includes soft tissues, brain, thyroid, and other cancers.

© 2024. Manfredi L. et al. Published online at http://www.aginganddisease.org/EN/10.14336/AD.2024.1166

SUPPLEMENTARY DATA

Supplementary Table 2. Distribution of the 10 cancer groups in men by the Relative Index of Inequality (RII) tertiles.

RII	1	2	3	Total
**Cancer groups**	**N (%)**	**N (%)**	**N (%)**	**N (%)**
Mouth and Larynx	24 (26.37%)	29 (31.87%)	38 (41.76%)	91 (100.00%)
Colorectal	109 (28.99%)	108 (28.72%)	159 (42.29%)	376 (100.00%)
Liver and Pancreas	46 (28.57%)	51 (31.68%)	64 (39.75%)	161 (100.00%)
Leukemia and Lymphoma	49 (26.20%)	61 (32.62%)	77 (41.18%)	187 (100.00%)
Melanoma	75 (26.22%)	89 (31.12%)	122 (42.66%)	286 (100.00%)
Lung	61 (22.76%)	69 (25.75%)	138 (51.49%)	268 (100.00%)
Prostate	267 (29.28%)	269 (29.50%)	376 (41.23%)	912 (100.00%)
Digestive Tract	28 (23.33%)	37 (30.83%)	55 (45.83%)	120 (100.00%)
Bladder and Kidneys	86 (29.35%)	77 (26.28%)	130 (44.37%)	293 (100.00%)
Other Cancers	47 (22.93%)	55 (26.83%)	103 (50.24%)	205 (100.00%)

Digestive tract includes stomach, esophagus, duodenum, and other digestive cancers, while other cancers includes soft tissues, brain, thyroid, and other cancers.

Supplementary Table 3. Distribution of the women sample across countries by age group.

Age class	30-39	40-49	50-59	60-69	70-80	Total (N, %)
**Country**	**N (%)**	**N (%)**	**N (%)**	**N (%)**	**N (%)**	**N (%)**
Italy	5 (0.21%)	27 (1.13%)	102 (4.26%)	58 (2.42%)	6 (0.25%)	198 (8.27%)
Spain	8 (0.33%)	66 (2.76%)	111 (4.63%)	50 (2.09%)	0 (0.00%)	235 (9.81%)
UK	1 (0.04%)	17 (0.71%)	47 (1.96%)	87 (3.63%)	79 (3.30%)	231 (9.65%)
Netherlands	0 (0.00%)	16 (0.67%)	143 (5.97%)	171 (7.14%)	0 (0.00%)	330 (13.78%)
Germany	4 (0.17%)	9 (0.38%)	50 (2.09%)	49 (2.05%)	0 (0.00%)	112 (4.68%)
Sweden	4 (0.17%)	56 (2.34%)	195 (8.14%)	240 (10.02%)	74 (3.09%)	569 (23.76%)
Denmark	0 (0.00%)	0 (0.00%)	432 (18.04%)	288 (12.03%)	0 (0.00%)	720 (30.06%)
**Total**	22 (0.92%)	191 (7.97%)	1080 (45.09%)	943 (39.37%)	159 (6.64%)	2395 (100.00%)

Supplementary Table 4. Distribution of the men sample across countries by age group

Age Class	20-29	30-39	40-49	50-59	60-69	70-80	Total
**Country**	**(N, %)**	**(N, %)**	**(N, %)**	**(N, %)**	**(N, %)**	**(N, %)**	**(N, %)**
Italy	0 (0.00%)	2 (0.05%)	41 (1.06%)	102 (2.64%)	57 (1.48%)	0 (0.00%)	202 (5.23%)
Spain	0 (0.00%)	1 (0.03%)	133 (3.45%)	235 (6.09%)	153 (3.96%)	0 (0.00%)	522 (13.53%)
UK	0 (0.00%)	1 (0.03%)	19 (0.49%)	72 (1.87%)	164 (4.25%)	94 (2.44%)	350 (9.07%)
Netherlands	0 (0.00%)	2 (0.05%)	31 (0.80%)	81 (2.10%)	19 (0.49%)	0 (0.00%)	133 (3.45%)
Germany	0 (0.00%)	0 (0.00%)	30 (0.78%)	127 (3.29%)	99 (2.57%)	0 (0.00%)	256 (6.63%)
Sweden	1 (0.03%)	11 (0.29%)	87 (2.25%)	405 (10.49%)	504 (13.06%)	98 (2.54%)	1106 (28.66%)
Denmark	0 (0.00%)	0 (0.00%)	0 (0.00%)	768 (19.90%)	522 (13.53%)	0 (0.00%)	1290 (33.43%)
**Total**	1 (0.03%)	17 (0.44%)	341 (8.84%)	1790 (46.39%)	1518 (39.34%)	192 (4.98%)	3859 (100.00%)

Supplementary Table 5. Proportion of Inertia (defined as Variance) Explained for Each Factor and Cumulative Inertia, women sample.

	Dim. 1	Dim. 2	Dim. 3	Dim. 4	Dim. 5	Dim. 6	Dim. 7	Dim. 8
**Variance**	0.096	0.078	0.076	0.068	0.067	0.066	0.066	0.065
**% of var.**	9.609	7.788	7.625	6.841	6.694	6.618	6.589	6.541
**Cumulative % of var.**	9.609	17.397	25.021	31.863	38.557	45.175	51.764	58.305
	**Dim. 9**	**Dim. 10**	**Dim. 11**	**Dim. 12**	**Dim. 13**	**Dim. 14**	**Dim. 15**	**Dim. 16**
**Variance**	0.065	0.064	0.064	0.063	0.063	0.062	0.032	0.003
**% of var.**	6.487	6.443	6.385	6.342	6.306	6.246	3.237	0.256
**Cumulative % of var.**	64.783	71.226	77.612	83.954	90.260	96.506	99.744	100.000

SUPPLEMENTARY DATA

Supplementary Table 6. Proportion of Inertia (defined as Variance) Explained for Each Factor and Cumulative Inertia, men sample.

	Dim. 1	Dim. 2	Dim. 3	Dim. 4	Dim. 5	Dim. 6	Dim. 7
**Variance**	0.113	0.097	0.094	0.084	0.083	0.083	0.081
**% of var.**	11.334	9.690	9.429	8.422	8.309	8.275	8.128
**Cumulative % of var.**	11.334	21.024	30.454	38.876	47.185	55.459	63.588
	**Dim. 8**	**Dim. 9**	**Dim. 10**	**Dim. 11**	**Dim. 12**	**Dim. 13**	
**Variance**	0.080	0.079	0.079	0.077	0.045	0.004	
**% of var.**	7.998	7.931	7.883	7.685	4.489	0.427	
**Cumulative % of var.**	71.586	79.516	87.399	95.084	99.573	100.000	

Supplementary Table 7. Distribution of the Relative Index of Inequality (RII) tertiles in each women cluster.

	1	2	3	4	5	6	Total
**RII**	**N (%)**	**N (%)**	**N (%)**	**N (%)**	**N (%)**	**N (%)**	**N (%)**
**1**	114 (25.11%)	176 (20.71%)	62 (26.72%)	27 (17.20%)	138 (26.19%)	55 (31.4%)	572 (23.88%)
**2**	149 (32.82%)	284 (33.41%)	73 (31.47%)	50 (31.85%)	156 (29.60%)	49 (28.0%)	761 (31.77%)
**3**	191 (42.07%)	390 (45.88%)	97 (41.81%)	80 (50.96%)	233 (44.21%)	71 (40.5%)	1062 (44.34%)
**Total**	454	850	232	157	527	175	2395

Cluster labels: 1 = "Breast cancer cluster"; 2 = "Type 2 Diabetes (T2D) and Coronary Heart Diseases (CHDs) cluster"; 3 = "Colorectal cancer cluster"; 4 = "Other cancers cluster"; 5 = "Cardiovascular Diseases (CVDs) group"; 6 = "Melanoma cluster".

Supplementary Table 8. Distribution of the Relative Index of Inequality (RII) tertiles in each men cluster.

	1	2	3	4	5	Total
**RII**	**N (%)**	**N (%)**	**N (%)**	**N (%)**	**N (%)**	**N (%)**
**1**	356 (26.29%)	86 (29.35%)	204 (29.44%)	109 (28.99%)	253 (22.13%)	1008 (26.12%)
**2**	385 (28.43%)	77 (26.28%)	208 (30.01%)	108 (28.72%)	391 (34.21%)	1169 (30.29%)
**3**	613 (45.27%)	130 (44.37%)	281 (40.55%)	159 (42.29%)	499 (43.66%)	1682 (43.59%)
**Totale**	1354	293	693	376	1143	3859

Cluster labels: 1 = "Cardiovascular Diseases (CVDs) cluster"; 2 = "Bladder and kidney cancer cluster"; 3 = "Prostate cancer cluster"; 4 = "Colorectal cancer cluster"; 5 = "Type 2 Diabetes (T2D) cluster".

Supplementary Table 9. Distribution of cancer types by Relative Index of Inequality (RII) in women with cancer.

Cancer type	RII = 1	RII = 2	RII = 3
**C509**	15.3	17.04	13.30
**C504**	9.54	9.11	8.38
**C508**	5.60	4.18	4.57
**C569**	4.21	3.84	4.40
**C502**	3.73	3.24	3.17
**C539**	3.08	4.27	2.60
**C421**	3.10	2.92	3.00
**C501**	3.08	2.69	2.97
**C541**	2.92	2.60	2.73
**C209**	2.87	2.40	2.60
**C187**	2.59	2.37	2.71
**C349**	1.57	2.65	3.39
**C447**	2.48	2.56	2.30
**C809**	1.90	1.78	1.60
**C505**	1.76	1.90	1.49
**C341**	1.46	1.58	1.95
**C779**	1.27	1.44	1.92
**C549**	1.74	1.10	1.77
**C739**	1.07	1.67	1.84

© 2024. Manfredi L. et al. Published online at http://www.aginganddisease.org/EN/10.14336/AD.2024.1166

**C503**	1.55	1.35	1.62
**C649**	1.23	1.58	1.57
**C445**	1.81	1.35	0.96
**C443**	1.48	1.16	1.22
**C446**	1.53	1.26	0.85
**C180**	1.04	0.98	1.42
**C679**	1.30	0.69	1.16
**C543**	0.88	1.23	0.81
**C182**	0.93	0.71	1.14
**C343**	0.88	0.78	1.05
**C700**	0.90	0.82	0.96
**C250**	0.81	0.62	0.81
**C199**	0.72	0.66	0.77
**C259**	0.67	0.53	0.85
**C169**	0.30	0.55	0.66
**C184**	0.67	0.39	0.39
**C186**	0.42	0.41	0.46
**C189**	0.30	0.55	0.39
**C220**	0.32	0.46	0.42
**C531**	0.37	0.30	0.35
**C163**	0.28	0.21	0.52
**C449**	0.23	0.32	0.42
**C239**	0.21	0.27	0.48
**C719**	0.35	0.25	0.33
**C340**	0.21	0.23	0.44
**C712**	0.21	0.30	0.33
**C500**	0.28	0.25	0.28
**C770**	0.25	0.34	0.20
**C185**	0.21	0.16	0.39
**C530**	0.16	0.21	0.39
**C711**	0.21	0.23	0.31
**C778**	0.21	0.30	0.24
**C519**	0.19	0.18	0.33
**C160**	0.14	0.30	0.24
**C183**	0.23	0.16	0.26
**C155**	0.16	0.27	0.20
**C492**	0.14	0.18	0.31
**C444**	0.09	0.23	0.28
**C342**	0.14	0.21	0.24
**C559**	0.25	0.21	0.13
**C693**	0.21	0.23	0.15
**C713**	0.3	0.05	0.24
**C162**	0.16	0.25	0.13
**C570**	0.19	0.21	0.13
**C710**	0.12	0.25	0.15
**C348**	0.16	0.18	0.17
**C659**	0.19	0.25	0.07
**C672**	0.07	0.27	0.17
**C240**	0.21	0.14	0.15
**C718**	0.19	0.21	0.09
**C251**	0.19	0.09	0.20
**C211**	0.16	0.18	0.13
**C762**	0.09	0.07	0.31
**C774**	0.16	0.21	0.09
**C099**	0.07	0.23	0.15
**C159**	0.14	0.11	0.17
**C181**	0.16	0.16	0.09
**C678**	0.19	0.11	0.11
**C241**	0.19	0.16	0.04

© 2024. Manfredi L. et al. Published online at http://www.aginganddisease.org/EN/10.14336/AD.2024.1166

**C709**	0.12	0.14	0.13
**C448**	0.14	0.11	0.13
**C168**	0.12	0.16	0.09
**C538**	0.09	0.16	0.11
**C221**	0.09	0.11	0.15
**C511**	0.19	0.07	0.07
**C725**	0.12	0.09	0.11
**C379**	0.07	0.11	0.13
**C384**	0.02	0.16	0.13
**C506**	0.07	0.11	0.13
**C210**	0.14	0.05	0.11
**C252**	0.09	0.14	0.07
**C772**	0.14	0.09	0.07
**C481**	0.14	0.02	0.13
**C258**	0.07	0.14	0.07
**C440**	0.12	0.05	0.11
**C480**	0.16	0.05	0.07
**C676**	0.12	0.07	0.09
**C669**	0.09	0.09	0.09
**C165**	0.12	0.09	0.04
**C172**	0.07	0.09	0.09
**C720**	0.02	0.16	0.07
**C773**	0.09	0.11	0.04
**C179**	0.07	0.07	0.09
**C249**	0.05	0.05	0.13
**C300**	0.05	0.07	0.11
**C482**	0.07	0.09	0.07
**C320**	0.05	0.09	0.07
**C548**	0.05	0.07	0.09
**C079**	0.00	0.05	0.15
**C188**	0.07	0.09	0.04
**C494**	0.02	0.11	0.07
**C542**	0.09	0.07	0.04
**C724**	0.07	0.09	0.04
**C154**	0.09	0.09	0.02
**C441**	0.05	0.05	0.09
**C321**	0.00	0.07	0.11
**C422**	0.05	0.09	0.04
**C529**	0	0.09	0.09
**C701**	0.02	0.07	0.09
**C729**	0.02	0.09	0.07
**C420**	0.05	0.05	0.07
**C499**	0.05	0.05	0.07
**C574**	0.05	0.05	0.07
**C009**	0.00	0.07	0.09
**C109**	0.02	0.14	0.00
**C164**	0.09	0.07	0.00
**C170**	0.09	0.05	0.02
**C510**	0.12	0.00	0.04
**C670**	0.02	0.07	0.07
**C714**	0.02	0.05	0.09
**C021**	0.05	0.05	0.04
**C069**	0.02	0.05	0.07
**C119**	0.07	0.05	0.02
**C402**	0.02	0.05	0.07
**C491**	0.00	0.00	0.13
**C717**	0.09	0.00	0.04
**C001**	0.07	0.05	0.00
**C019**	0.05	0.05	0.02

© 2024. Manfredi L. et al. Published online at http://www.aginganddisease.org/EN/10.14336/AD.2024.1166

**C049**	0.05	0.05	0.02
**C161**	0.05	0.00	0.07
**C171**	0.05	0.05	0.02
**C030**	0.02	0.02	0.07
**C089**	0.02	0.07	0.02
**C269**	0.07	0.00	0.04
**C495**	0.02	0.07	0.02
**C674**	0.02	0.02	0.07
**C579**	0.09	0.00	0.02
**C060**	0.00	0.02	0.07
**C254**	0.07	0.02	0.00
**C518**	0.00	0.07	0.02
**C029**	0.00	0.05	0.04
**C310**	0.05	0.02	0.02
**C413**	0.05	0.00	0.04
**C715**	0.05	0.00	0.04
**C763**	0.00	0.05	0.04
**C031**	0.02	0.02	0.04
**C716**	0.02	0.02	0.04
**C771**	0.02	0.02	0.04
**C023**	0.00	0.00	0.07
**C051**	0.00	0.00	0.07
**C496**	0.05	0.00	0.02
**C512**	0.02	0.05	0.00
**C000**	0.02	0.00	0.04
**C111**	0.02	0.02	0.02
**C166**	0.02	0.02	0.02
**C329**	0.00	0.02	0.04
**C412**	0.02	0.00	0.04
**C442**	0.00	0.02	0.04
**C699**	0.02	0.02	0.02
**C740**	0.00	0.02	0.04
**C749**	0.00	0.02	0.04
**C068**	0.00	0.05	0.00
**C311**	0.00	0.05	0.00
**C694**	0.00	0.05	0.00
**C020**	0.00	0.00	0.04
**C052**	0.02	0.02	0.00
**C253**	0.02	0.02	0.00
**C260**	0.02	0.02	0.00
**C339**	0.02	0.02	0.00
**C398**	0.00	0.00	0.04
**C410**	0.02	0.02	0.00
**C414**	0.00	0.02	0.02
**C423**	0.02	0.02	0.00
**C424**	0.02	0.02	0.00
**C493**	0.00	0.00	0.04
**C540**	0.00	0.00	0.04
**C673**	0.00	0.02	0.02
**C680**	0.02	0.00	0.02
**C689**	0.02	0.00	0.02
**C761**	0.00	0.00	0.04
**C764**	0.00	0.02	0.02
**C022**	0.02	0.00	0.00
**C028**	0.00	0.02	0.00
**C039**	0.00	0.00	0.02
**C040**	0.00	0.02	0.00
**C041**	0.00	0.00	0.02
**C050**	0.00	0.02	0.00

© 2024. Manfredi L. et al. Published online at http://www.aginganddisease.org/EN/10.14336/AD.2024.1166

**C059**	0.00	0.00	0.02
**C061**	0.00	0.02	0.00
**C090**	0.00	0.02	0.00
**C102**	0.00	0.02	0.00
**C103**	0.00	0.02	0.00
**C108**	0.00	0.00	0.02
**C112**	0.00	0.02	0.00
**C113**	0.02	0.00	0.00
**C129**	0.00	0.00	0.02
**C138**	0.00	0.00	0.02
**C139**	0.00	0.02	0.00
**C150**	0.00	0.00	0.02
**C151**	0.00	0.00	0.02
**C153**	0.00	0.02	0.00
**C178**	0.00	0.00	0.02
**C212**	0.00	0.00	0.02
**C218**	0.00	0.00	0.02
**C268**	0.02	0.00	0.00
**C322**	0.00	0.02	0.00
**C328**	0.00	0.02	0.00
**C383**	0.02	0.00	0.00
**C400**	0.00	0.02	0.00
**C471**	0.00	0.02	0.00
**C490**	0.00	0.00	0.02
**C578**	0.02	0.00	0.00
**C589**	0.00	0.02	0.00
**C675**	0.02	0.00	0.00
**C677**	0.00	0.00	0.02
**C688**	0.02	0.00	0.00
**C690**	0.02	0.00	0.00
**C723**	0.00	0.00	0.02
**C750**	0.00	0.02	0.00
**C760**	0.00	0.02	0.00
**C765**	0.00	0.02	0.00
**C767**	0.02	0.00	0.00

RII tertiles: RII = 1 (High Socioeconomic Position (SEP)), RII = 2 (Medium SEP), RII = 3 (Low SEP). C509: Malignant neoplasm of the breast, unspecified; C504: Malignant neoplasm of the lower-inner quadrant of the breast; C508: Malignant neoplasm of overlapping lesions of the breast; C569: Malignant neoplasm of the ovary, unspecified; C502: Malignant neoplasm of the upper-inner quadrant of the breast; C539: Malignant neoplasm, unspecified; C421: Malignant neoplasm of the nasopharynx; C501: Malignant neoplasm of the nipple and areola; C541: Malignant neoplasm of the uterus; C209: Malignant neoplasm of unspecified part of the digestive system; C187: Malignant neoplasm of the cecum; C349: Malignant neoplasm of the pleura; C447: Malignant neoplasm of the skin of the face; C809: Malignant neoplasm, unspecified; C505: Malignant neoplasm of the axillary tail of the breast; C341: Malignant neoplasm of the left bronchus; C779: Malignant neoplasm of unspecified site; C549: Malignant neoplasm of the mammary gland; C739: Malignant neoplasm of the testis; C503: Malignant neoplasm of the upper-outer quadrant of the breast; C649: Malignant neoplasm of skin in other and unspecified parts of the face; C445: Malignant neoplasm of skin of the scalp; C443: Malignant neoplasm of skin of lower limb; C446: Malignant neoplasm of skin in other and unspecified parts of the body; C180: Malignant neoplasm of the rectum; C679: Malignant neoplasm of the eye, unspecified site; C543: Malignant neoplasm of the pancreas; C182: Malignant neoplasm of the larynx; C343: Malignant neoplasm of bronchus; C700: Malignant neoplasm in head and neck region; C250: Malignant neoplasm of pancreas, unspecified site; C199: Malignant neoplasm of colon, unspecified part; C259: Malignant neoplasm of other endocrine glands, unspecified site; C169: Malignant neoplasm of adrenal gland, unspecified site; C184: Malignant neoplasm of kidney, unspecified site; C186: Malignant neoplasm of thyroid gland, unspecified site; C189: Malignant neoplasm of stomach, unspecified part; C220: Malignant neoplasm of gallbladder, unspecified site; C531: Malignant neoplasm of cervix uteri (cervical cancer); C163: Malignant neoplasm of pancreas (specific type); C449: Malignant neoplasm of skin in extremities, unspecified site; C239: Malignant neoplasm of liver, unspecified part; C719: Malignant neoplasm of ear, unspecified site; C340: Malignant neoplasm of trachea, unspecified site; C712: Malignant neoplasm of lung, unspecified site; C500: Malignant neoplasm of breast (specific classification); C770: Secondary malignant tumors in respiratory system (if applicable); C185: Malignant neoplasm of small intestine, unspecified part; and C530: Malignant neoplasm of thymus; C711: Malignant neoplasm of the right bronchus; C778: Malignant neoplasm of other and unspecified parts of the respiratory system; C519: Malignant neoplasm of the prostate; C160: Malignant neoplasm of the liver; C183: Malignant neoplasm of the colon, unspecified; C155: Malignant neoplasm of the pleura; C492: Malignant neoplasm of skin in other and unspecified parts of the body; C444: Malignant neoplasm of the skin of the scalp; C342: Malignant neoplasm of the bronchus; C559: Malignant neoplasm of the thymus; C693: Malignant neoplasm of the skin of the eyelid; C713: Malignant neoplasm of the respiratory system; C162: Malignant neoplasm of the pancreas; C570: Malignant neoplasm of the uterus; C710: Malignant neoplasm of the chest wall; C348: Malignant neoplasm of soft tissue of the head and neck; C659: Malignant neoplasm of kidney, unspecified; C672: Malignant neoplasm of skin in other parts of the body; C240: Malignant neoplasm of rectum; C718: Malignant neoplasm of trachea, unspecified site; C251: Malignant neoplasm of breast, unspecified site; C211: Malignant neoplasm of salivary gland; C762: Malignant neoplasm of pancreas, unspecified site; C774: Malignant neoplasm of gallbladder, unspecified site; C099: Malignant neoplasm of nose, unspecified site; C159: Malignant neoplasm of ovary, unspecified site; C181: Malignant neoplasm of stomach, unspecified part; C678: Malignant neoplasm of skin in extremities; C241: Malignant neoplasm of liver, unspecified part; C709: Malignant neoplasm of pancreas, unspecified site; C448: Malignant neoplasm of

skin on face; C168: Malignant neoplasm of stomach, unspecified part; C538: Malignant neoplasm of pancreas, unspecified site; C221: Malignant neoplasm of larynx, unspecified site; C511: Malignant neoplasm of skin on chest; C725: Malignant neoplasm in head and neck region, unspecified site; C379: Malignant neoplasm in lung, unspecified site; C384: Malignant neoplasm in larynx, unspecified site; C506: Malignant neoplasm in breast, unspecified site; C210: Malignant neoplasm in prostate, unspecified site; C252: Malignant neoplasm in pancreas, unspecified site; C772: Malignant neoplasm in lung, unspecified site; C481: Malignant neoplasm in skin in extremities, unspecified site; C258: Malignant neoplasm in liver, unspecified part; C440: Malignant neoplasm in skin, unspecified site; C480: Malignant neoplasm in pancreas, unspecified site; C676: Malignant neoplasm in head and neck region, unspecified site; C669: Malignant neoplasm in chest, unspecified site; C165: Malignant neoplasm in kidney; C172: Malignant neoplasm of the bone; C720: Malignant neoplasm of the base of the tongue; C773: Malignant neoplasm of the respiratory system; C179: Malignant neoplasm of the rectum, unspecified; C249: Malignant neoplasm of the pancreas, unspecified; C300: Malignant neoplasm of the larynx; C482: Malignant neoplasm of the pancreas, unspecified; C320: Malignant neoplasm of the bronchus; C548: Malignant neoplasm of the salivary glands; C079: Malignant neoplasm of the thyroid gland; C188: Malignant neoplasm of the colon; C494: Malignant neoplasm of the skin of the extremities; C542: Malignant neoplasm of the uterus; C724: Malignant neoplasm of the brain; C154: Malignant neoplasm of the pleura; C441: Malignant neoplasm of the skin of the face; C321: Malignant neoplasm of the tongue; C422: Malignant neoplasm of the stomach; C529: Malignant neoplasm of the bladder; C701: Malignant neoplasm of the head and neck region; C729: Malignant neoplasm of soft tissue, unspecified site; C420: Malignant neoplasm of the neck; C499: Malignant neoplasm of skin in extremities, unspecified site; C574: Malignant neoplasm of pancreas, unspecified site; C009: Malignant neoplasm of mouth, unspecified site; C109: Malignant neoplasm of pancreas, unspecified site; C164: Malignant neoplasm of kidney, unspecified site; C170: Malignant neoplasm of larynx, unspecified site; C510: Malignant neoplasm of breast, unspecified site; C670: Malignant neoplasm of salivary glands, unspecified site; C714: Malignant neoplasm of tongue, unspecified site; C021: Malignant neoplasm of tongue, unspecified site; C069: Malignant neoplasm of throat, unspecified site; C119: Malignant neoplasm of nose, unspecified site; C402: Malignant neoplasm of respiratory system, unspecified site; C491: Malignant neoplasm of skin in extremities, unspecified site; C717: Malignant neoplasm of liver, unspecified part; C001: Malignant neoplasm of mouth, unspecified site; C019: Malignant neoplasm of face, unspecified site; C049: Malignant neoplasm of ear, unspecified site; C161: Malignant neoplasm of thyroid gland, unspecified site; C171: Malignant neoplasm of kidney, unspecified site; C030: Malignant neoplasm of salivary glands, unspecified site; C089: Malignant neoplasm of face, unspecified site; C269: Malignant neoplasm of pancreas, unspecified site; C495: Malignant neoplasm of skin on face, unspecified site; C674: Malignant neoplasm in head and neck region, unspecified site; C579: Malignant neoplasm of cervix uteri (cervical cancer); C060: Malignant neoplasm in skin in extremities, unspecified site; C254: Malignant neoplasm in skin in extremities, unspecified site; C518: Malignant neoplasm in breast; C029: Malignant neoplasm of the tongue, unspecified; C310: Malignant neoplasm of the larynx; C413: Malignant neoplasm of the hypopharynx; C715: Malignant neoplasm of the thyroid; C763: Malignant neoplasm of the pancreas; C031: Malignant neoplasm of the tongue; C716: Malignant neoplasm of the head and neck; C771: Malignant neoplasm of the skin, unspecified site; C023: Malignant neoplasm of the lip; C051: Malignant neoplasm of the tongue; C496: Malignant neoplasm of the pancreas, unspecified site; C512: Malignant neoplasm of the skin, unspecified site; C000: Malignant neoplasm of the lip, unspecified site; C111: Malignant neoplasm of the tongue; C166: Malignant neoplasm of the liver; C329: Malignant neoplasm of the bladder, unspecified site; C412: Malignant neoplasm of the larynx, unspecified site; C442: Malignant neoplasm of skin on the scalp; C699: Malignant neoplasm of the brain, unspecified site; C740: Malignant neoplasm of respiratory system, unspecified site; C749: Malignant neoplasm of soft tissue, unspecified site; C068: Malignant neoplasm of throat, unspecified site; C311: Malignant neoplasm of pharynx, unspecified site; C694: Malignant neoplasm of respiratory system, unspecified site; C020: Malignant neoplasm of pancreas, unspecified site; C052: Malignant neoplasm of tongue, unspecified site; C253: Malignant neoplasm of pancreas, unspecified site; C260: Malignant neoplasm of skin, unspecified site; C339: Malignant neoplasm of respiratory system, unspecified site; C398: Malignant neoplasm of neck, unspecified site; C410: Malignant neoplasm of breast, unspecified site; C414: Malignant neoplasm of breast, unspecified site; C423: Malignant neoplasm of pancreas, unspecified site; C424: Malignant neoplasm of mouth, unspecified site; C493: Malignant neoplasm of respiratory system, unspecified site; C540: Malignant neoplasm of ovary, unspecified site; C673: Malignant neoplasm of eye, unspecified site; C680: Malignant neoplasm of brain, unspecified site; C689: Malignant neoplasm of skin, unspecified site; C761: Malignant neoplasm of pancreas, unspecified site; C764: Malignant neoplasm of respiratory system, unspecified site; C022: Malignant neoplasm of eye, unspecified site; C028: Malignant neoplasm of lip, unspecified site; C039: Malignant neoplasm of pancreas, unspecified site; C040: Malignant neoplasm of throat, unspecified site; C041: Malignant neoplasm of esophagus, unspecified site; C050: Malignant neoplasm of larynx, unspecified site; C059: Malignant neoplasm of tongue, unspecified site; C061: Malignant neoplasm of skin on head and neck region; C090: Malignant neoplasm of nose; C102: Malignant neoplasm of the tongue; C103: Malignant neoplasm of the mouth; C108: Malignant neoplasm of the cheek; C112: Malignant neoplasm of the oropharynx; C113: Malignant neoplasm of the soft palate; C129: Malignant neoplasm of the larynx; C138: Malignant neoplasm of the skin, unspecified site; C139: Malignant neoplasm of the neck, unspecified site; C150: Malignant neoplasm of the stomach; C151: Malignant neoplasm of the rectum; C153: Malignant neoplasm of the pancreas, unspecified site; C178: Malignant neoplasm of the liver; C212: Malignant neoplasm of the kidney; C218: Malignant neoplasm of the urinary bladder; C268: Malignant neoplasm of the colon; C322: Malignant neoplasm of the bronchi; C328: Malignant neoplasm of the chest wall; C383: Malignant neoplasm of the salivary glands; C400: Malignant neoplasm of the pancreas, unspecified site; C471: Malignant neoplasm of the oral cavity, unspecified site; C490: Malignant neoplasm of the breast, unspecified site; C578: Malignant neoplasm of the ovary, unspecified site; C589: Malignant neoplasm of the respiratory system, unspecified site; C675: Malignant neoplasm of the lung, unspecified site; C677: Malignant neoplasm of the pancreas, unspecified site; C688: Malignant neoplasm of the stomach, unspecified part; C690: Malignant neoplasm of the tongue, unspecified site; C723: Malignant neoplasm of head and neck region, unspecified site; C750: Malignant neoplasm of pancreas, unspecified site; C760: Malignant neoplasm in throat, unspecified site; C765: Malignant neoplasm of skin, unspecified site; C767: Malignant neoplasm of face, unspecified site. Codes from the ICD-10 classification system.

Supplementary Table 10. Distribution of cancer types by Relative Index of Inequality (RII) in men with cancer.

Cancer type	RII = 1	RII = 2	RII = 3
**C619**	35.84	29.88	30.54
**C209**	4.84	5.12	4.24
**C421**	4.11	4.61	4.21
**C679**	4.18	4.16	4.38
**C349**	2.81	3.25	5.25
**C187**	2.61	3.74	3.17
**C341**	1.84	3.22	4.05
**C649**	3.21	2.25	2.49
**C445**	2.94	2.84	1.94

**C779**	1.80	2.18	1.94
**C809**	1.50	1.84	2.00
**C443**	1.44	1.21	1.48
**C446**	1.60	1.28	1.09
**C343**	0.80	1.32	1.39
**C250**	1.00	1.00	1.12
**C180**	1.20	0.93	0.96
**C199**	1.04	1.07	0.88
**C182**	0.90	0.90	1.01
**C447**	1.10	0.83	0.79
**C169**	0.77	1.00	0.85
**C320**	0.67	0.90	0.96
**C259**	0.87	0.83	0.77
**C160**	0.70	1.04	0.68
**C220**	0.67	0.87	0.82
**C384**	0.23	0.62	1.09
**C159**	0.53	0.69	0.68
**C672**	0.53	0.69	0.68
**C184**	0.84	0.48	0.55
**C155**	0.40	0.66	0.63
**C186**	0.47	0.66	0.52
**C678**	0.43	0.73	0.49
**C189**	0.63	0.38	0.63
**C442**	0.53	0.59	0.49
**C449**	0.60	0.45	0.49
**C340**	0.27	0.52	0.74
**C444**	0.47	0.48	0.46
**C739**	0.53	0.31	0.46
**C629**	0.30	0.69	0.30
**C719**	0.43	0.28	0.52
**C448**	0.57	0.35	0.25
**C163**	0.23	0.35	0.52
**C770**	0.27	0.42	0.33
**C321**	0.30	0.48	0.22
**C712**	0.47	0.31	0.19
**C700**	0.37	0.28	0.30
**C713**	0.30	0.31	0.30
**C183**	0.37	0.28	0.25
**C342**	0.27	0.21	0.41
**C099**	0.37	0.07	0.44
**C778**	0.20	0.38	0.27
**C329**	0.13	0.42	0.30
**C185**	0.27	0.28	0.27
**C240**	0.17	0.38	0.25
**C348**	0.13	0.31	0.36
**C711**	0.43	0.14	0.22
**C001**	0.07	0.24	0.46
**C710**	0.13	0.42	0.19
**C251**	0.20	0.28	0.25
**C718**	0.20	0.31	0.22
**C492**	0.23	0.24	0.22
**C162**	0.23	0.17	0.27
**C168**	0.20	0.24	0.16
**C659**	0.23	0.21	0.14
**C154**	0.13	0.17	0.27
**C221**	0.20	0.21	0.14
**C693**	0.20	0.17	0.14
**C300**	0.10	0.17	0.19
**C774**	0.07	0.28	0.11

© 2024. Manfredi L. et al. Published online at http://www.aginganddisease.org/EN/10.14336/AD.2024.1166

**C676**	0.20	0.17	0.08
**C772**	0.20	0.14	0.11
**C129**	0.07	0.21	0.11
**C139**	0.13	0.10	0.16
**C239**	0.17	0.07	0.14
**C674**	0.17	0.10	0.11
**C161**	0.13	0.10	0.14
**C188**	0.13	0.10	0.14
**C724**	0.13	0.21	0.03
**C670**	0.17	0.07	0.11
**C181**	0.10	0.10	0.14
**C601**	0.07	0.14	0.11
**C021**	0.13	0.10	0.08
**C079**	0.23	0.03	0.05
**C210**	0.07	0.21	0.03
**C029**	0.13	0.14	0.03
**C252**	0.10	0.14	0.05
**C509**	0.07	0.03	0.19
**C714**	0.13	0.00	0.16
**C069**	0.10	0.07	0.11
**C109**	0.03	0.14	0.11
**C241**	0.10	0.10	0.08
**C441**	0.10	0.10	0.08
**C621**	0.13	0.10	0.05
**C675**	0.13	0.10	0.05
**C720**	0.03	0.14	0.11
**C480**	0.17	0.07	0.03
**C019**	0.07	0.14	0.05
**C725**	0.03	0.07	0.16
**C119**	0.07	0.07	0.11
**C170**	0.07	0.07	0.11
**C031**	0.07	0.14	0.03
**C165**	0.03	0.07	0.14
**C420**	0.13	0.07	0.03
**C762**	0.03	0.17	0.03
**C062**	0.10	0.07	0.05
**C164**	0.00	0.14	0.08
**C172**	0.07	0.10	0.05
**C049**	0.10	0.03	0.08
**C249**	0.07	0.03	0.11
**C491**	0.13	0.03	0.05
**C609**	0.13	0.03	0.05
**C009**	0.03	0.03	0.14
**C090**	0.07	0.07	0.05
**C494**	0.00	0.07	0.11
**C771**	0.03	0.07	0.08
**C328**	0.07	0.07	0.03
**C493**	0.07	0.07	0.03
**C495**	0.07	0.07	0.03
**C669**	0.00	0.03	0.14
**C179**	0.03	0.10	0.03
**C258**	0.13	0.03	0.00
**C153**	0.10	0.00	0.05
**C211**	0.07	0.00	0.08
**C402**	0.07	0.03	0.05
**C440**	0.10	0.00	0.05
**C600**	0.00	0.10	0.05
**C716**	0.03	0.07	0.05
**C760**	0.10	0.00	0.05

© 2024. Manfredi L. et al. Published online at http://www.aginganddisease.org/EN/10.14336/AD.2024.1166

**C773**	0.07	0.00	0.08
**C051**	0.07	0.07	0.00
**C148**	0.07	0.07	0.00
**C310**	0.07	0.07	0.00
**C422**	0.07	0.07	0.00
**C717**	0.00	0.03	0.11
**C068**	0.03	0.07	0.03
**C166**	0.03	0.07	0.03
**C501**	0.03	0.07	0.03
**C040**	0.00	0.07	0.05
**C269**	0.07	0.00	0.05
**C689**	0.03	0.03	0.05
**C100**	0.00	0.07	0.03
**C138**	0.07	0.03	0.00
**C140**	0.00	0.07	0.03
**C379**	0.03	0.07	0.00
**C410**	0.03	0.07	0.00
**C423**	0.03	0.07	0.00
**C729**	0.07	0.03	0.00
**C319**	0.03	0.03	0.03
**C499**	0.03	0.03	0.03
**C631**	0.03	0.03	0.03
**C060**	0.00	0.03	0.05
**C158**	0.00	0.03	0.05
**C268**	0.00	0.00	0.08
**C398**	0.00	0.00	0.08
**C414**	0.00	0.03	0.05
**C424**	0.03	0.00	0.05
**C671**	0.00	0.03	0.05
**C709**	0.03	0.00	0.05
**C767**	0.00	0.03	0.05
**C383**	0.00	0.07	0.00
**C413**	0.07	0.00	0.00
**C490**	0.07	0.00	0.00
**C632**	0.00	0.07	0.00
**C715**	0.00	0.07	0.00
**C024**	0.03	0.03	0.00
**C039**	0.03	0.00	0.03
**C059**	0.03	0.00	0.03
**C098**	0.00	0.03	0.03
**C130**	0.00	0.03	0.03
**C150**	0.00	0.03	0.03
**C171**	0.03	0.00	0.03
**C400**	0.00	0.03	0.03
**C412**	0.03	0.00	0.03
**C419**	0.00	0.03	0.03
**C602**	0.03	0.03	0.00
**C673**	0.03	0.00	0.03
**C699**	0.03	0.03	0.00
**C740**	0.03	0.03	0.00
**C763**	0.03	0.03	0.00
**C637**	0.00	0.00	0.05
**C004**	0.03	0.00	0.00
**C008**	0.00	0.00	0.03
**C020**	0.00	0.03	0.00
**C030**	0.03	0.00	0.00
**C048**	0.03	0.00	0.00
**C081**	0.00	0.00	0.03
**C091**	0.03	0.00	0.00

© 2024. Manfredi L. et al. Published online at http://www.aginganddisease.org/EN/10.14336/AD.2024.1166

**C102**	0.00	0.03	0.00
**C108**	0.03	0.00	0.00
**C110**	0.00	0.03	0.00
**C118**	0.00	0.00	0.03
**C131**	0.00	0.00	0.03
**C132**	0.03	0.00	0.00
**C152**	0.03	0.00	0.00
**C178**	0.00	0.00	0.03
**C218**	0.00	0.00	0.03
**C253**	0.03	0.00	0.00
**C254**	0.03	0.00	0.00
**C313**	0.00	0.03	0.00
**C339**	0.03	0.00	0.00
**C380**	0.00	0.03	0.00
**C381**	0.03	0.00	0.00
**C403**	0.00	0.00	0.03
**C479**	0.00	0.00	0.03
**C481**	0.00	0.03	0.00
**C482**	0.00	0.00	0.03
**C488**	0.03	0.00	0.00
**C496**	0.00	0.00	0.03
**C498**	0.03	0.00	0.00
**C500**	0.00	0.03	0.00
**C504**	0.00	0.03	0.00
**C620**	0.00	0.03	0.00
**C677**	0.03	0.00	0.00
**C680**	0.00	0.00	0.03
**C688**	0.03	0.00	0.00
**C690**	0.00	0.03	0.00
**C694**	0.00	0.03	0.00
**C695**	0.00	0.00	0.03
**C701**	0.00	0.00	0.03
**C728**	0.00	0.03	0.00
**C761**	0.00	0.03	0.00
**C765**	0.00	0.00	0.03

RII tertiles: RII = 1 (High Socioeconomic Position (SEP)), RII = 2 (Medium SEP), RII = 3 (Low SEP). C619: Malignant neoplasm of the prostate; C209: Malignant neoplasm of the bronchus and lung; C421: Malignant neoplasm of the blood and bone marrow; C679: Malignant neoplasm of the bladder, unspecified; C349: Malignant neoplasm of the lung; C187: Malignant neoplasm of the sigmoid colon; C341: Malignant neoplasm of the upper lobe, bronchus, or lung; C649: Malignant neoplasm of the kidney; C445: Malignant neoplasm of the skin of other parts of the trunk; C779: Secondary malignant neoplasm of lymph nodes, unspecified; C809: Malignant neoplasm, unspecified; C443: Malignant neoplasm of the skin of unspecified parts; C446: Malignant neoplasm of the skin of the lower limb; C343: Malignant neoplasm of the lower lobe, bronchus, or lung; C250: Malignant neoplasm of the pancreas, head; C180: Malignant neoplasm of the colon; C199: Malignant neoplasm of the intestine, unspecified; C182: Malignant neoplasm of the ascending colon; C447: Malignant neoplasm of the skin of the upper limb; C169: Malignant neoplasm of the stomach; C320: Malignant neoplasm of the glottis; C259: Malignant neoplasm of the endocrine pancreas; C160: Malignant neoplasm of the esophagus; C220: Malignant neoplasm of the liver; C384: Malignant neoplasm of the pleura; C159: Malignant neoplasm of the pharynx, unspecified; C672: Malignant neoplasm of the ureter; C184: Malignant neoplasm of the transverse colon; C155: Malignant neoplasm of the lower third of the esophagus; C186: Malignant neoplasm of the descending colon; C678: Malignant neoplasm of the bladder; C189: Malignant neoplasm of the kidney, unspecified; C442: Malignant neoplasm of the skin of the upper limb; C449: Malignant neoplasm of skin, unspecified; C340: Malignant neoplasm of the main bronchus; C444: Malignant neoplasm of the skin of the scalp and neck; C739: Malignant neoplasm of the thyroid gland; C629: Malignant neoplasm of the ovary, unspecified; C719: Malignant neoplasm of the brain, unspecified; C448: Malignant neoplasm of the skin at other sites; C163: Malignant neoplasm of the diaphragm; C770: Secondary malignant neoplasms in lymph nodes in head, face, and neck; C321: Malignant neoplasm in subglottis; C712: Malignant neoplasm in brain; C700: Malignant neoplasms in brain stem; C713: Malignant tumor in cerebrum; C183: Malignant tumor in hepatic flexure; C342: Malignant tumor in middle lobe, bronchus, or lung; C099: Malignant tumor in tonsil; C778: Secondary malignant tumor in lymph nodes at other sites; C329: Malignant tumor in kidney; C185: Tumor in splenic flexure; C240: Tumor in extrahepatic bile duct; and C348: Tumor in lung at overlapping sites; C711: Malignant neoplasm of the brain, cerebellum; C001: Malignant neoplasm of the external lip; C710: Malignant neoplasm of the brain, unspecified; C251: Malignant neoplasm of the body of the pancreas; C718: Malignant neoplasm of the brain, overlapping sites; C492: Malignant neoplasm of the connective and soft tissue, unspecified; C162: Malignant neoplasm of the middle third of the esophagus; C168: Malignant neoplasm of the gastroesophageal junction; C659: Malignant neoplasm of the ovary; C154: Malignant neoplasm of the cervical esophagus; C221: Malignant neoplasm of the

gallbladder; C693: Malignant neoplasm of the orbit; C300: Malignant neoplasm of the nasal cavity; C774: Secondary malignant neoplasm of lymph nodes, other specified sites; C676: Malignant neoplasm of the bladder, trigone; C772: Secondary malignant neoplasm of lymph nodes, intra-abdominal; C129: Malignant neoplasm of the pharynx; C139: Malignant neoplasm of the head and neck, unspecified; C239: Malignant neoplasm of the pancreas, unspecified; C674: Malignant neoplasm of the urethra; C161: Malignant neoplasm of the upper third of the esophagus; C188: Malignant neoplasm of the rectosigmoid junction; C724: Malignant neoplasm of the spinal cord; C670: Malignant neoplasm of the renal pelvis; C181: Malignant neoplasm of the cecum; C601: Malignant neoplasm of the testis; C021: Malignant neoplasm of the tongue; C079: Malignant neoplasm of other and ill-defined sites in the respiratory system; C210: Malignant neoplasm of the rectum; C029: Malignant neoplasm of the tongue, unspecified; C252: Malignant neoplasm of the tail of the pancreas; and C509: Malignant neoplasm of the breast, unspecified; C714: Malignant neoplasm of the brain, frontal lobe; C069: Malignant neoplasm of the oropharynx; C109: Malignant neoplasm of the nasopharynx; C241: Malignant neoplasm of the extrahepatic bile ducts; C441: Malignant neoplasm of the skin of the trunk; C621: Malignant neoplasm of the testis, unspecified; C675: Malignant neoplasm of the bladder, ureter; C720: Malignant neoplasm of the spinal cord; C480: Malignant neoplasm of the retroperitoneum; C019: Malignant neoplasm of the mouth; C725: Malignant neoplasm of the cranial nerves; C119: Malignant neoplasm of the nasopharynx, overlapping lesions; C170: Malignant neoplasm of the small intestine; C031: Malignant neoplasm of the tongue; C165: Malignant neoplasm of the pancreas, body; C420: Malignant neoplasm of the blood; C762: Secondary malignant neoplasm of the thorax; C062: Malignant neoplasm of the sublingual gland; C164: Malignant neoplasm of the pancreas; C172: Malignant neoplasm of the colon, rectosigmoid junction; C049: Malignant neoplasm of the tongue, anterior; C249: Malignant neoplasm of the pancreas, unspecified; C491: Malignant neoplasm of the breast; C609: Malignant neoplasm of the prostate; C009: Malignant neoplasm of the lip; C090: Malignant neoplasm of the nose; C494: Malignant neoplasm of the pancreas; C771: Malignant neoplasm of lymph nodes; C328: Malignant neoplasm of the chest wall; C493: Malignant neoplasm of soft tissue; C495: Malignant neoplasm of skin; and C669: Malignant neoplasm of ureter; C179: Malignant neoplasm of the small intestine, unspecified; C258: Malignant neoplasm of the endocrine pancreas; C153: Malignant neoplasm of the upper third of the esophagus; C211: Malignant neoplasm of the rectosigmoid junction; C402: Malignant neoplasm of the connective and soft tissue of the upper limb; C440: Malignant neoplasm of the skin of unspecified sites; C600: Malignant neoplasm of the testis, unspecified; C716: Malignant neoplasm of the brain, parietal lobe; C760: Malignant neoplasm of overlapping sites of the thorax; C773: Secondary malignant neoplasm of lymph nodes, thoracic; C051: Malignant neoplasm of the soft palate; C148: Malignant neoplasm of the thoracic esophagus; C310: Malignant neoplasm of the nasal cavity and middle ear; C422: Malignant neoplasm of the blood and bone marrow, unspecified; C717: Malignant neoplasm of the brain, occipital lobe; C068: Malignant neoplasm of the major salivary glands; C166: Malignant neoplasm of the pancreas, overlapping lesion; C501: Malignant neoplasm of the breast, upper-outer quadrant; C040: Malignant neoplasm of the floor of the mouth; C269: Malignant neoplasm of the kidney, unspecified; C689: Malignant neoplasm of the urinary tract, unspecified; C100: Malignant neoplasm of the oral cavity; C138: Malignant neoplasm of the pharynx, overlapping sites; C140: Malignant neoplasm of the hypopharynx; C379: Malignant neoplasm of the respiratory system; C410: Malignant neoplasm of the connective and soft tissue of the lower limb; C423: Malignant neoplasm of the bone marrow, unspecified; C729: Malignant neoplasm of the spinal cord, unspecified; C319: Malignant neoplasm of the pharynx, unspecified; C499: Malignant neoplasm of soft tissue, unspecified; C631: Malignant neoplasm of the prostate; C060: Malignant neoplasm of the parotid gland; C158: Malignant neoplasm of the esophagus, overlapping lesion; C268: Malignant neoplasm of the small intestine, overlapping lesion; C398: Malignant neoplasm of large intestine, overlapping lesion; C414: Malignant neoplasm of peripheral nerves of upper limb; C424: Malignant neoplasm of peripheral nerves of lower limb; C671: Malignant neoplasm of ureter; C709: Malignant neoplasm of spinal cord, overlapping lesion; C767: Secondary malignant neoplasm in thorax, overlapping sites; C383: Malignant neoplasm in mediastinum; C413: Malignant neoplasm in connective and soft tissue in thorax; C490: Malignant neoplasm in connective and soft tissue in head, face, and neck; C632: Malignant neoplasm in testis, overlapping lesion; C715: Malignant neoplasm in brain, cerebellum; C024: Malignant neoplasm in tongue, dorsal surface; C039: Malignant neoplasm in tongue, overlapping lesion; C059: Malignant neoplasm in salivary glands; C098: Malignant neoplasm in pharynx, overlapping sites; C130: Malignant neoplasm in nasopharynx; C150: Malignant neoplasm in middle third of esophagus; C171: Malignant neoplasm in pancreas; C400: Malignant neoplasm in connective and soft tissue in upper limb; C412: Malignant neoplasm in connective and soft tissue in thorax; C419: Malignant neoplasm in connective and soft tissue, unspecified; and C602: Malignant neoplasm in testis, overlapping lesion; C673: Malignant neoplasm of the ureter; C699: Malignant neoplasm of the brain and other parts of the central nervous system, unspecified; C740: Malignant neoplasm of the adrenal gland, cortex; C763: Secondary malignant neoplasm of overlapping sites of the digestive system; C637: Malignant neoplasm of the spermatic cord; C004: Malignant neoplasm of the lip, vermilion border; C008: Malignant neoplasm of overlapping sites of the lip; C020: Malignant neoplasm of the tongue, base; C030: Malignant neoplasm of the gum, upper; C048: Malignant neoplasm of the floor of the mouth, overlapping lesion; C081: Malignant neoplasm of the salivary glands, parotid; C091: Malignant neoplasm of the tonsils; C102: Malignant neoplasm of the oropharynx, anterior wall; C108: Malignant neoplasm of the oropharynx, overlapping lesion; C110: Malignant neoplasm of the nasopharynx, superior wall; C118: Malignant neoplasm of the nasopharynx, overlapping lesion; C131: Malignant neoplasm of the hypopharynx, piriform sinus; C132: Malignant neoplasm of the hypopharynx, posterior wall; C152: Malignant neoplasm of the middle third of the esophagus; C178: Malignant neoplasm of the retroperitoneum; C218: Malignant neoplasm of the rectum; C253: Malignant neoplasm of the pancreas, tail; C254: Malignant neoplasm of the pancreas, overlapping lesion; C313: Malignant neoplasm of the nasal cavity; C339: Malignant neoplasm of the respiratory tract, unspecified; C380: Malignant neoplasm of the heart; C381: Malignant neoplasm of the mediastinum; C403: Malignant neoplasm of connective and soft tissue of the pelvis; C479: Malignant neoplasm of peripheral nerves and autonomic nervous system, unspecified; C481: Malignant neoplasm of retroperitoneum; C482: Malignant neoplasm of peritoneum; C488: Malignant neoplasm of overlapping sites of retroperitoneum and peritoneum; C496: Malignant neoplasm of connective and soft tissue of abdomen; C498: Malignant neoplasm of connective and soft tissue, unspecified; C500: Malignant neoplasm of breast, unspecified; C504: Malignant neoplasm of lower-inner quadrant of breast; C620: Malignant neoplasm of testis; C677: Malignant neoplasm of bladder, overlapping lesion; C680: Malignant neoplasm of urethra; C688: Malignant neoplasm of urinary tract, overlapping lesion; C690: Malignant neoplasm of eye, unspecified; C694: Malignant neoplasm of retina; C695: Malignant neoplasm of choroid; C701:

Malignant neoplasm of meninges, unspecified; C728: Malignant neoplasm of spinal cord and other parts of nervous system, overlapping lesion; C761: Secondary malignant neoplasm at unspecified sites; and C765: Secondary malignant neoplasm at overlapping sites in respiratory and intrathoracic organs. Codes from the ICD-10 classification system.

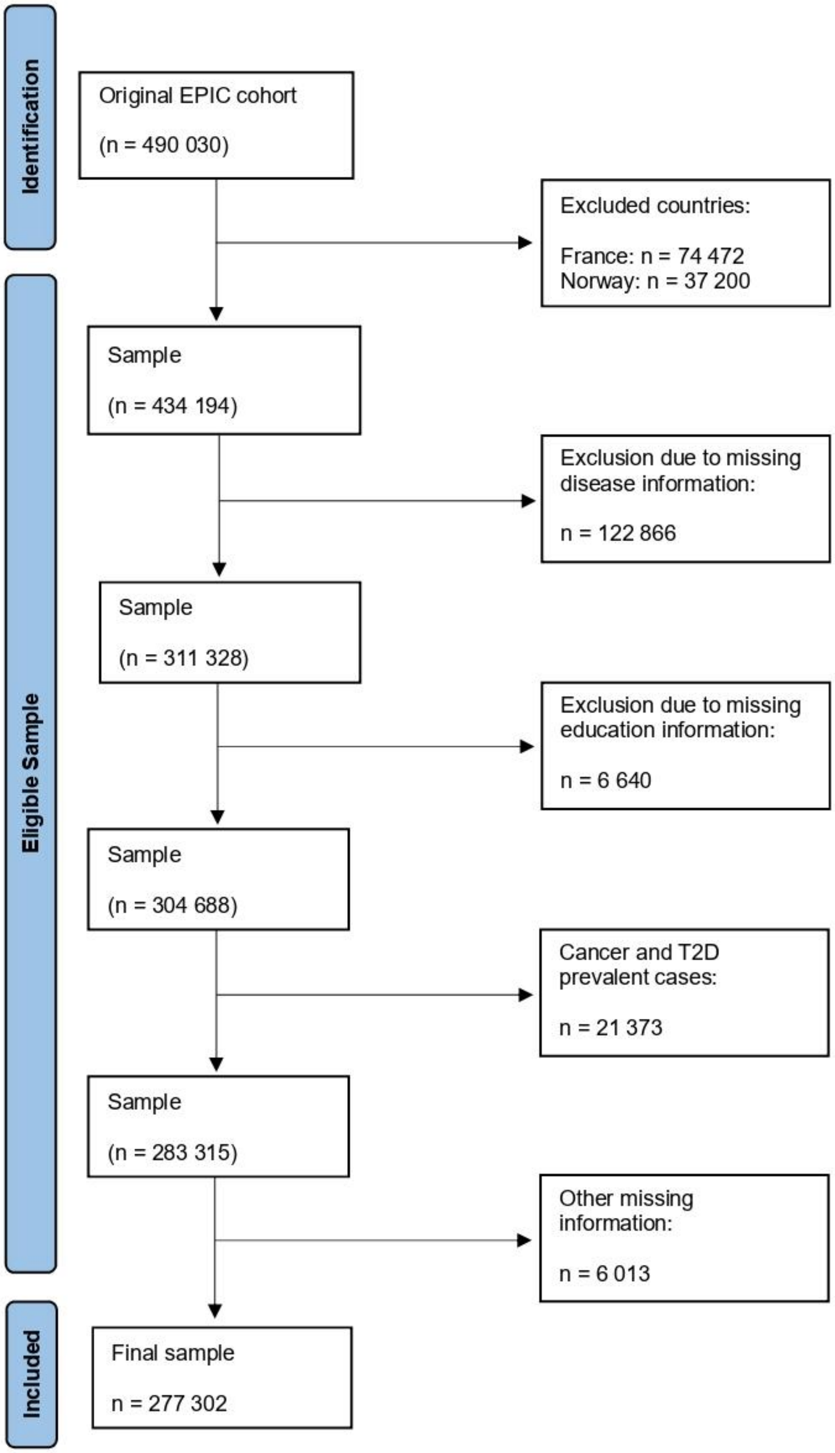

Supplementary Figure 1. Flow chart of the study. EPIC: European Prospective Investigation into Cancer and Nutrition; T2D: Type 2 Diabetes.

© 2024. Manfredi L. et al. Published online at http://www.aginganddisease.org/EN/10.14336/AD.2024.1166

SUPPLEMENTARY DATA

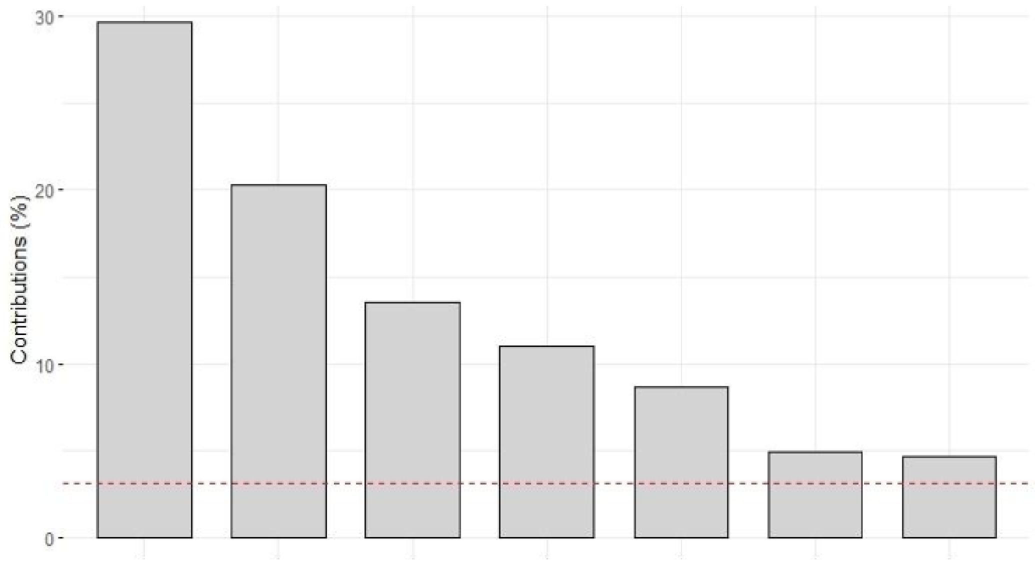

Supplementary Figure 2. Contribution of variables to Dim-1, women sample. Variables that have the major contribution to first factor inertia: No Type 2 Diabetes (T2D), T2D, Coronary heart disease (CHD), No CHD, Breast, No Stroke.

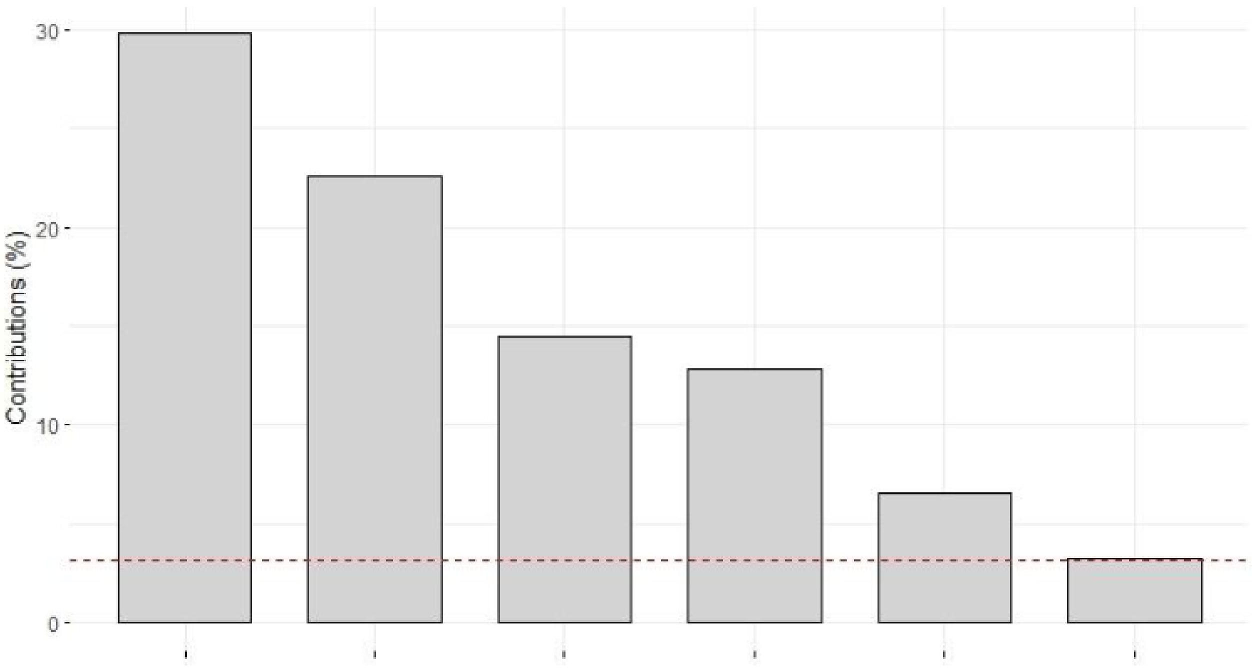

Supplementary Figure 3. Contribution of variables to Dim-2, women sample. Variables that have the major contribution to second factor inertia: Stroke, Coronary heart disease (CHD), No CHD, No Stroke, Lung, Melanoma.

© 2024. Manfredi L. et al. Published online at http://www.aginganddisease.org/EN/10.14336/AD.2024.1166

SUPPLEMENTARY DATA

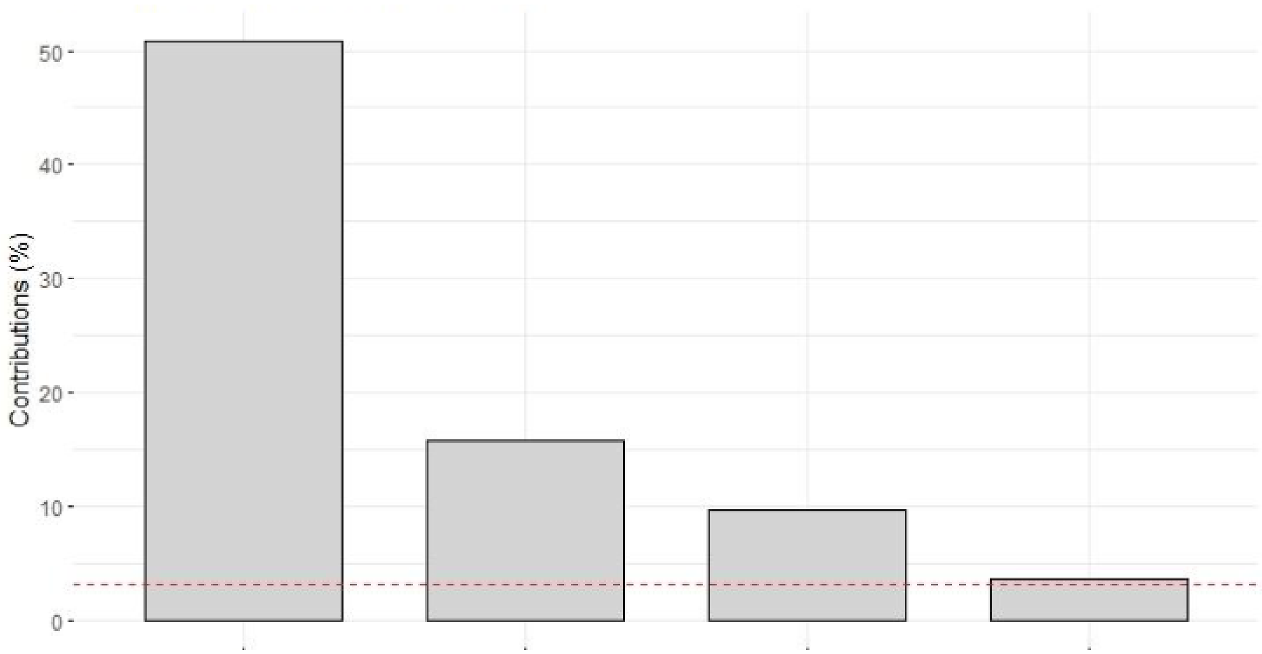

Supplementary Figure 4. Contribution of variables to Dim-3, women sample. Variables that have the major contribution to third factor inertia: Breast, No Breast, Colorectal, Body of the Uterus.

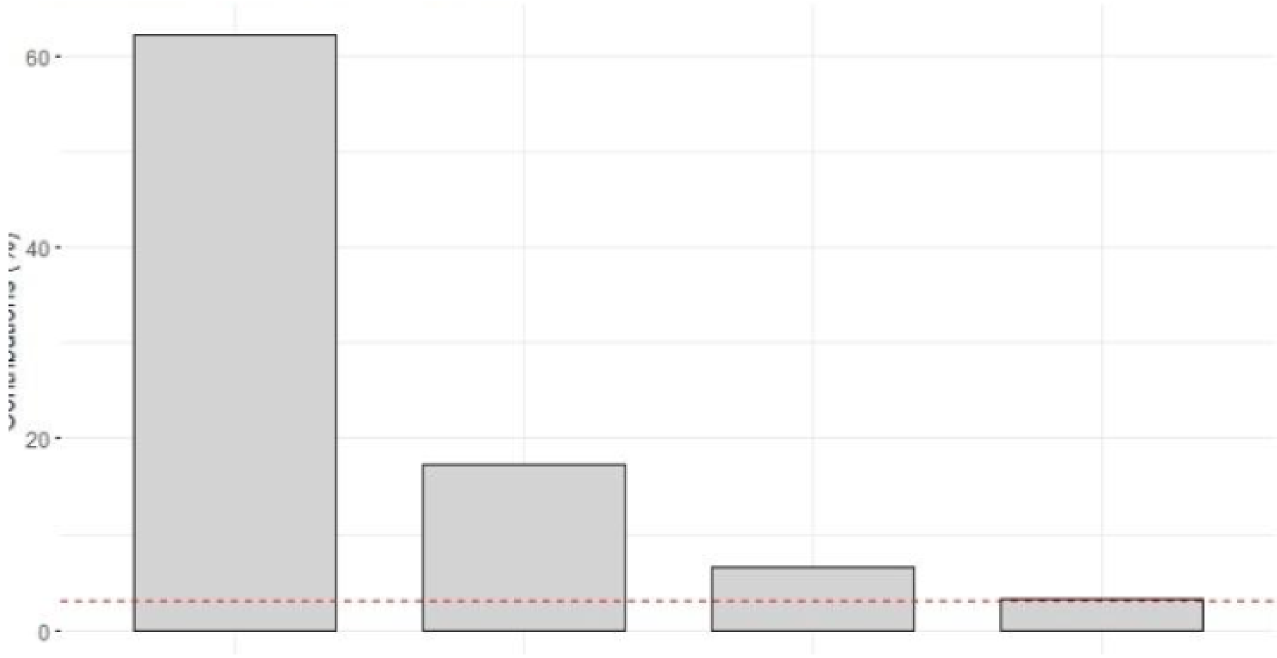

Supplementary Figure 5. Contribution of variables to Dim-4, women sample. Variables that have the major contribution to fourth factor inertia: Colorectal, Melanoma, No Colorectal, Other cancers.

© 2024. Manfredi L. et al. Published online at http://www.aginganddisease.org/EN/10.14336/AD.2024.1166

SUPPLEMENTARY DATA

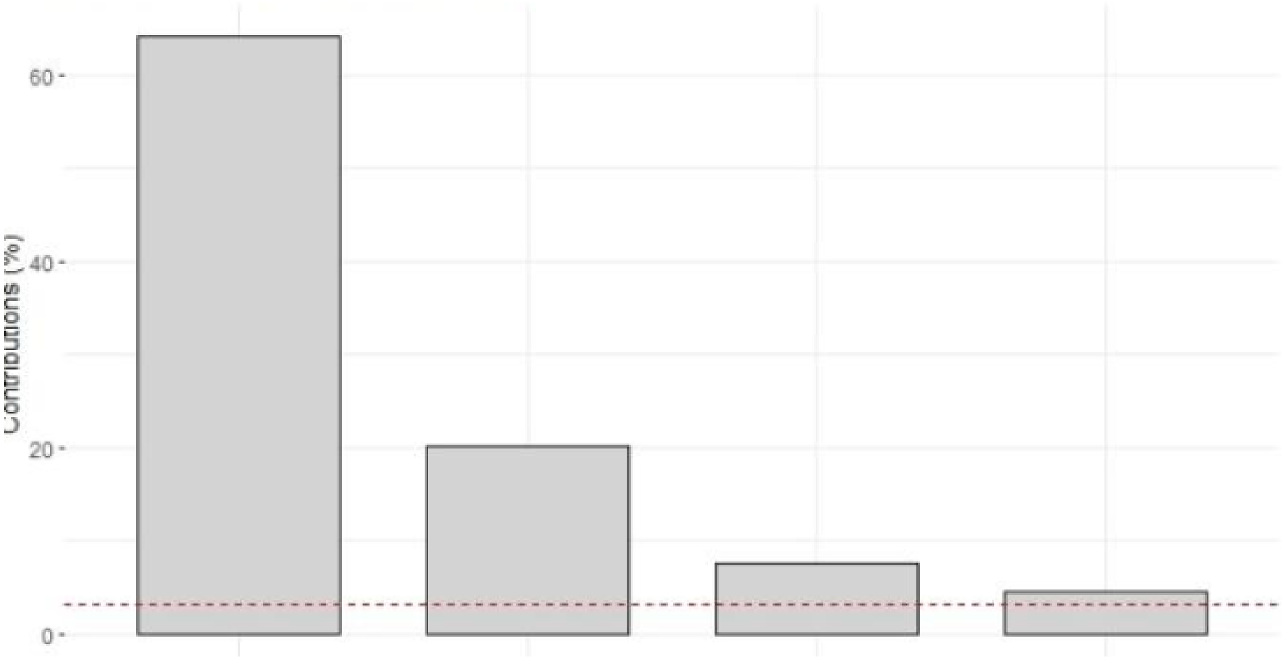

Supplementary Figure 6. Contribution of variables to Dim-5, women sample. Variables that have the major contribution to fifth factor inertia: Other cancers, Lung, Melanoma, No Other cancers.

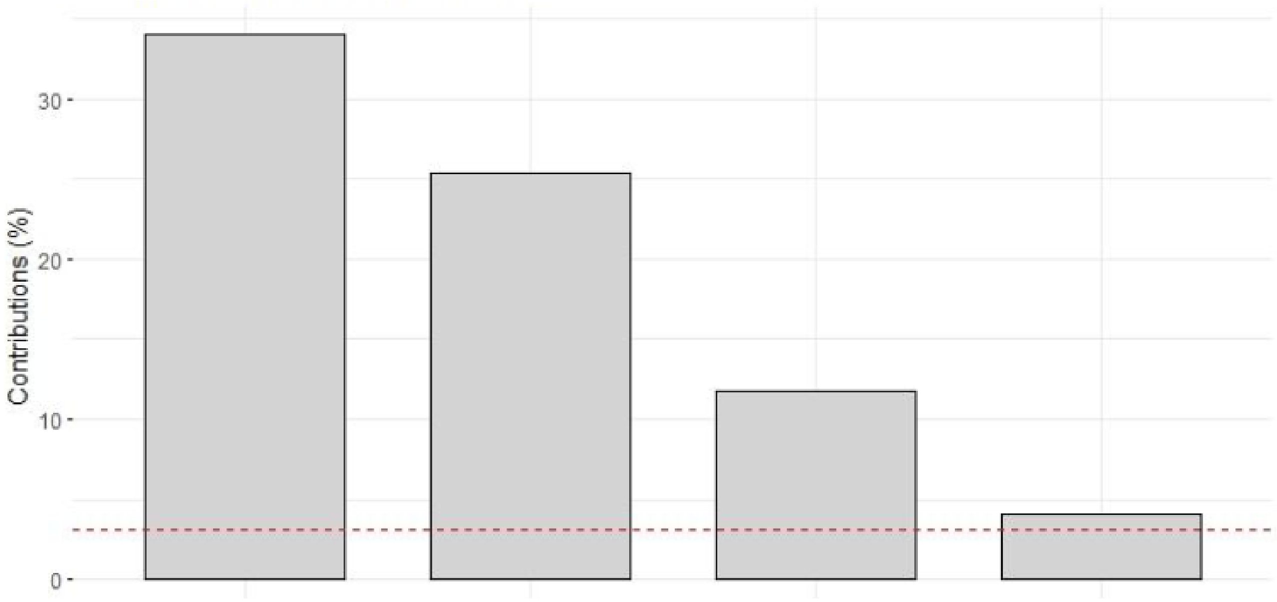

Supplementary Figure 7. Contribution of variables to Dim-6, women sample. Variables that have the major contribution to sixth factor inertia: Lung, Melanoma, Leukemia and Lymphoma, Body of the Uterus.

© 2024. Manfredi L. et al. Published online at http://www.aginganddisease.org/EN/10.14336/AD.2024.1166

SUPPLEMENTARY DATA

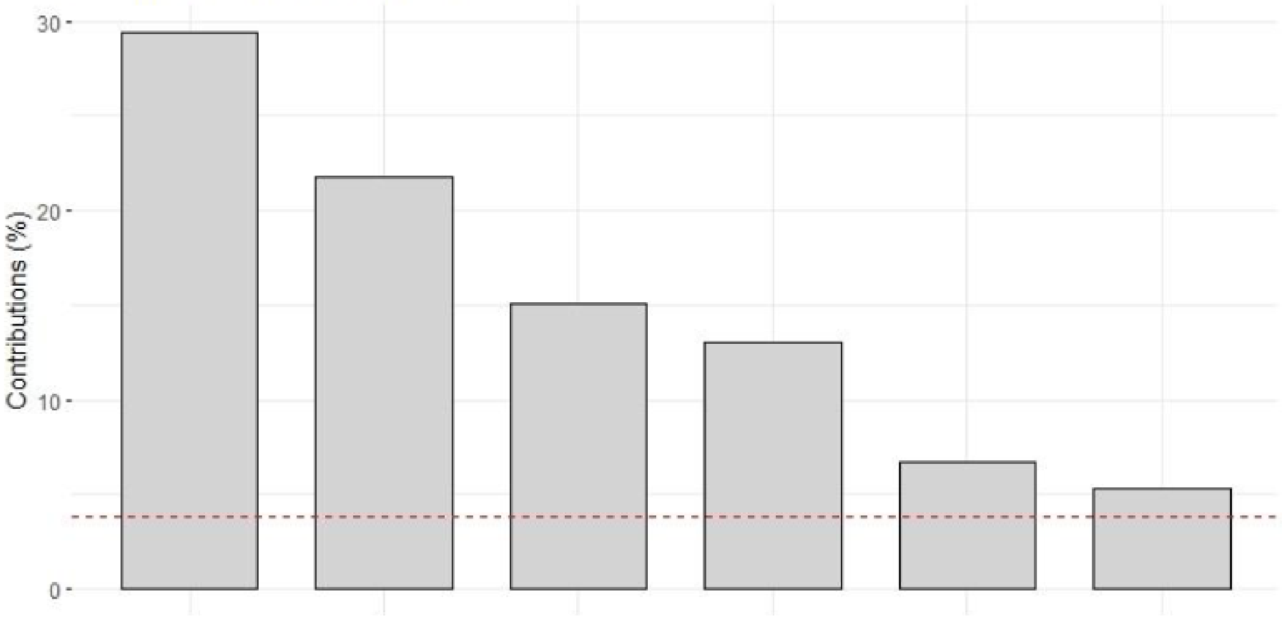

Supplementary Figure 8. Contribution of variables to Dim-1, men sample. Variables that have the major contribution to first factor inertia: No Type 2 Diabetes (T2D), T2D, Coronary heart disease (CHD), No CHD, Liver and Pancreas, Stroke.

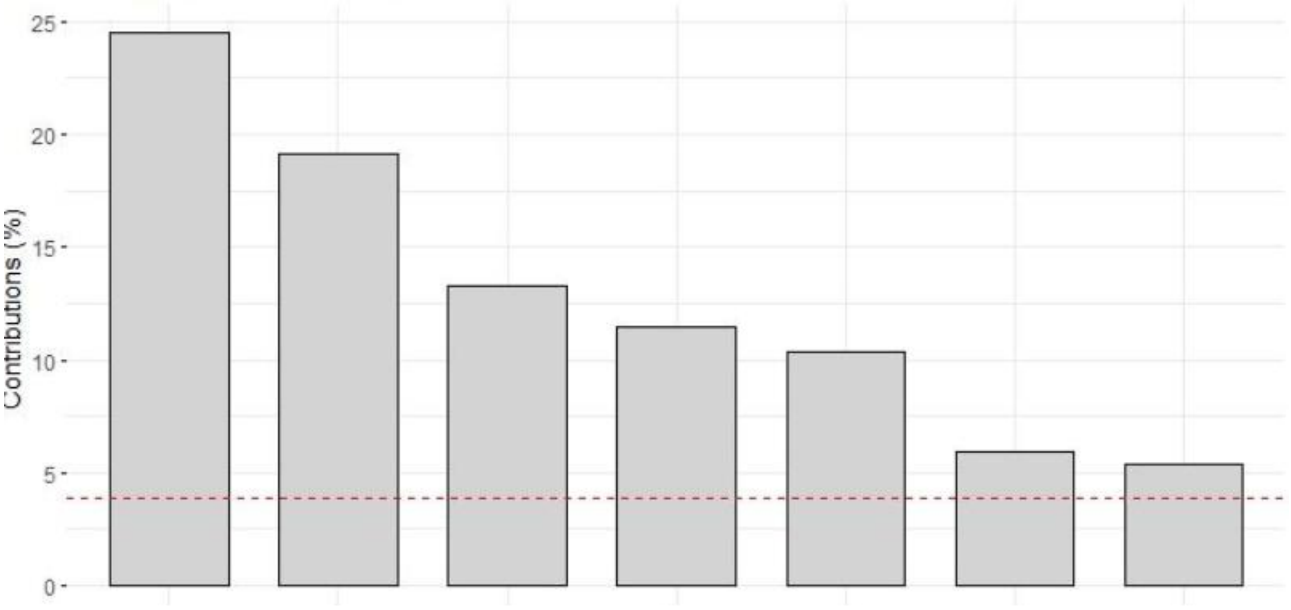

Supplementary Figure 9. Contribution of variables to Dim-2, men sample. Variables that have the major contribution to second factor inertia: Stroke, Prostate, Coronary heart disease (CHD), No CHD, No Stroke, No prostate, Leukaemia and Lymphoma.

© 2024. Manfredi L. et al. Published online at http://www.aginganddisease.org/EN/10.14336/AD.2024.1166

SUPPLEMENTARY DATA

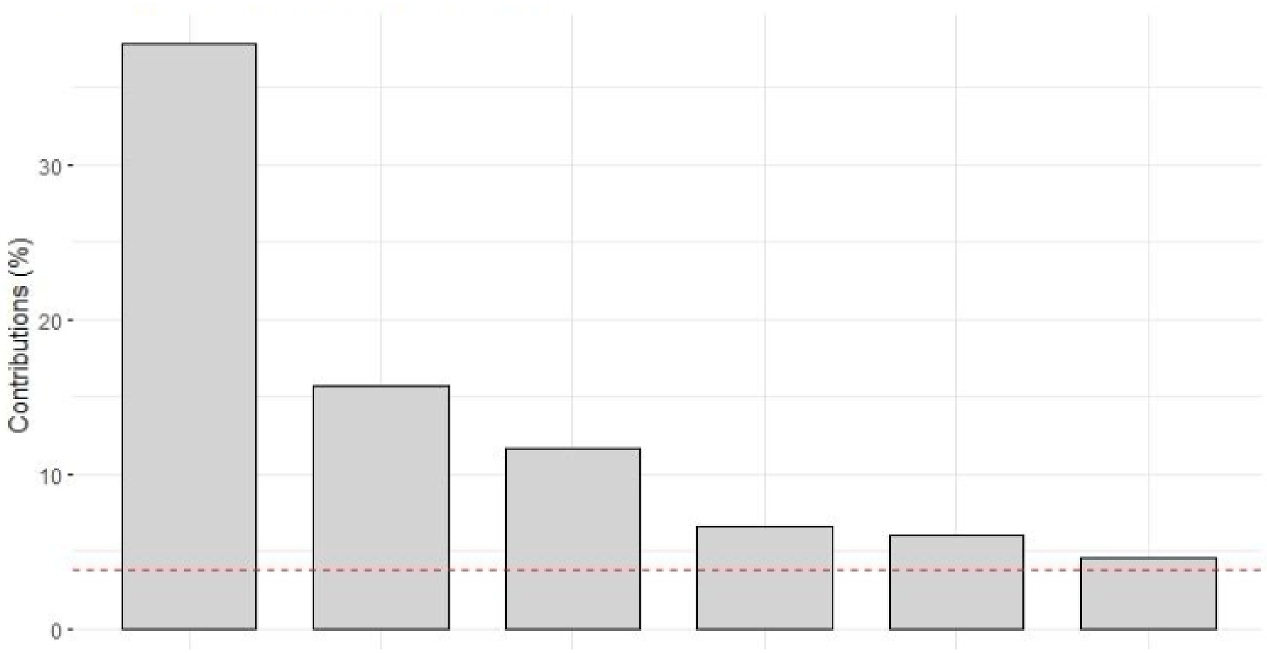

Supplementary Figure 10. Contribution of variables to Dim-3, men sample. Variables that have the major contribution to third factor inertia: Prostate, Stroke, No Prostate, No Stroke, Melanoma, Other cancers.

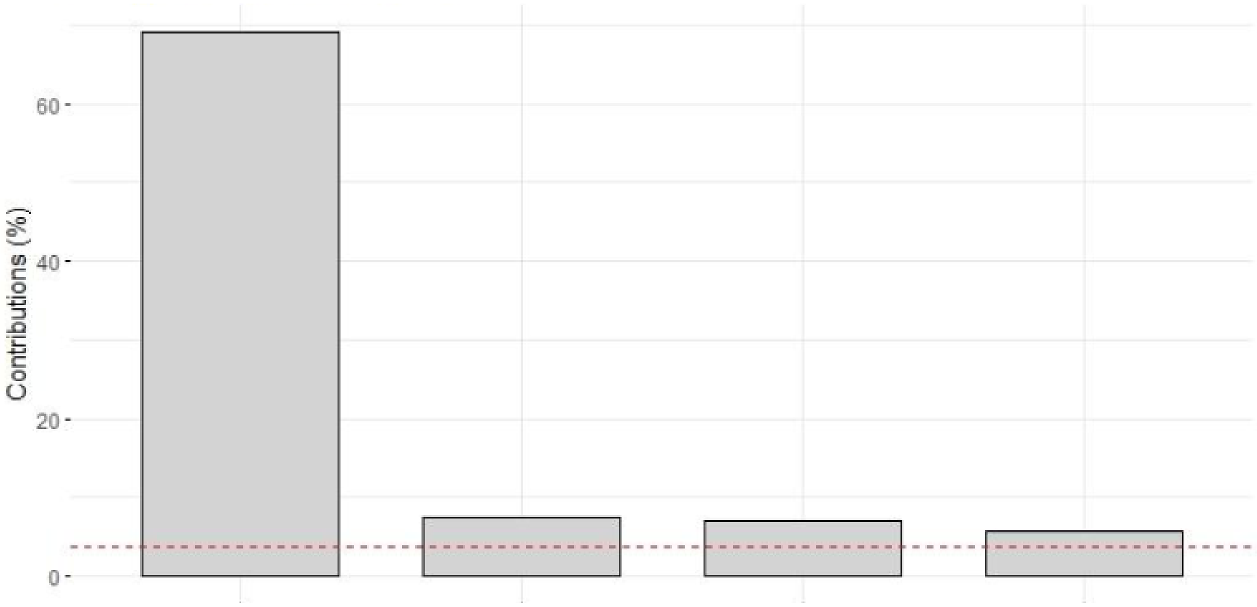

Supplementary Figure 11. Contribution of variables to Dim-4, men sample. Variables that have the major contribution to fourth factor inertia: Colorectal, No Colorectal, Liver and Pancreas, Bladder and Kidney.

© 2024. Manfredi L. et al. Published online at http://www.aginganddisease.org/EN/10.14336/AD.2024.1166

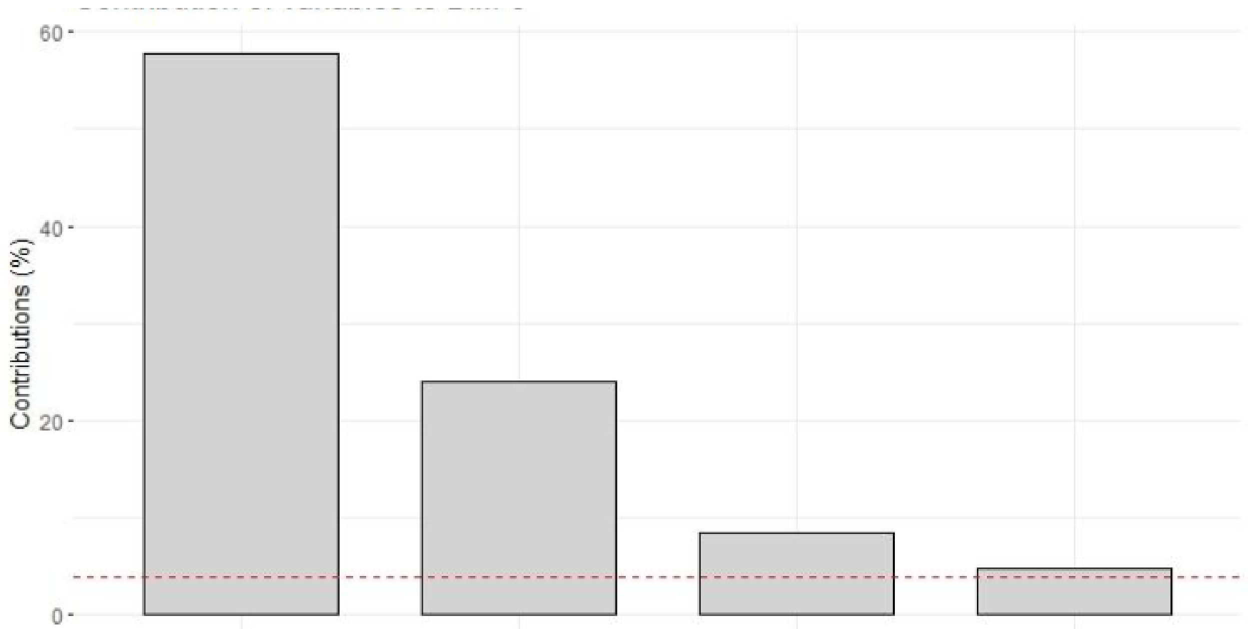

Supplementary Figure 12. Contribution of variables to Dim-5, men sample. Variables that have the major contribution to fifth factor inertia: Bladder and Kidney, Melanoma, Lung, No Bladder and Kidney.

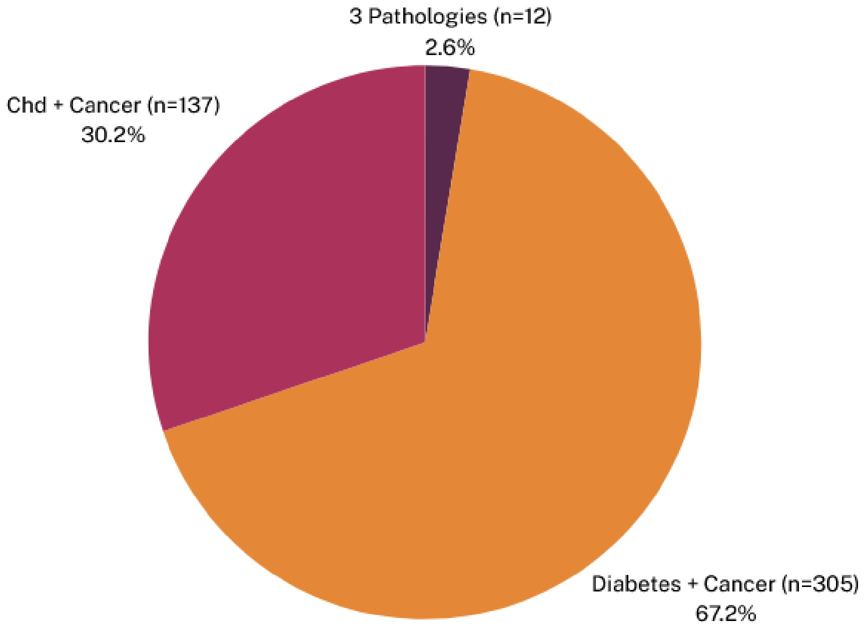

Cancer: Breast Cancer (454)
CHD: coronary heart disease.

Supplementary Figure 13. Cluster 1, women sample.

© 2024. Manfredi L. et al. Published online at http://www.aginganddisease.org/EN/10.14336/AD.2024.1166

SUPPLEMENTARY DATA

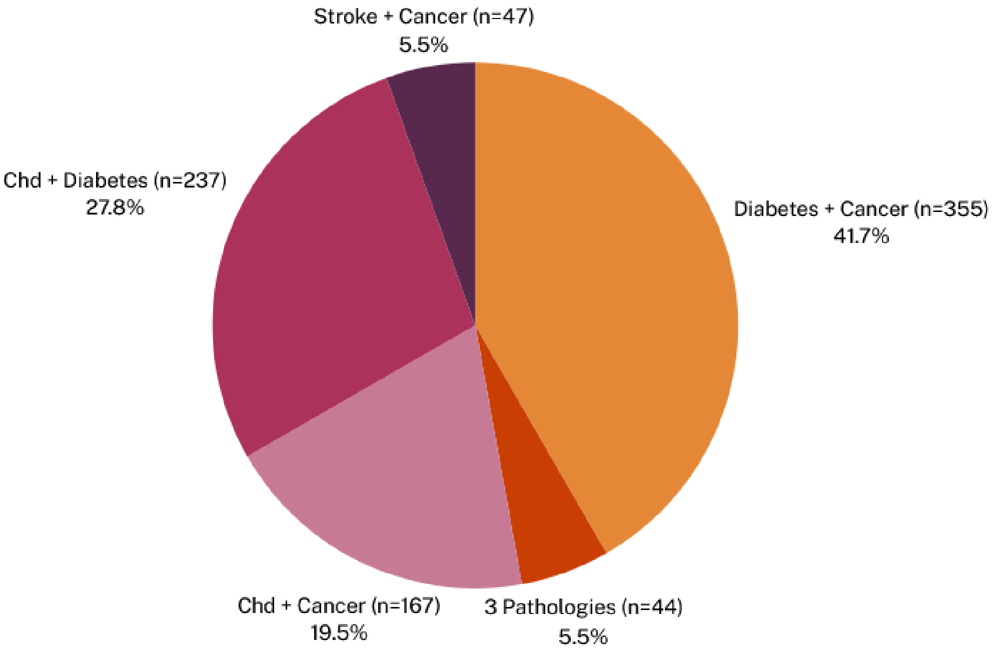

Supplementary Figure 14. Cluster 2, women sample.
Cancer: Lung cancer (166), Body of the uterus (90), Leukemia-Lymphoma (89), Liver-Pancreas (74), Bladder-Renal (57), Ovary-Other female genitalia (42), Cervix (31), Digestive-Tract (25), Mouth-Larynx (24)
CHD: coronary heart disease.

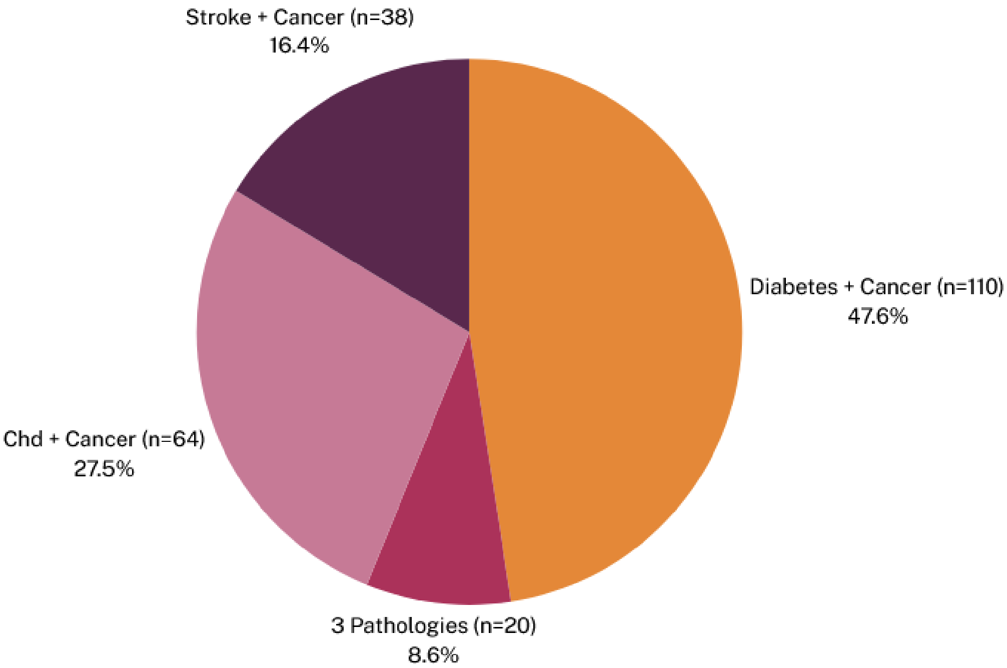

Supplementary Figure 15. Cluster 3, women sample.
Cancer: Colorectal Cancer (232)
CHD: coronary heart disease.

© 2024. Manfredi L. et al. Published online at http://www.aginganddisease.org/EN/10.14336/AD.2024.1166

SUPPLEMENTARY DATA

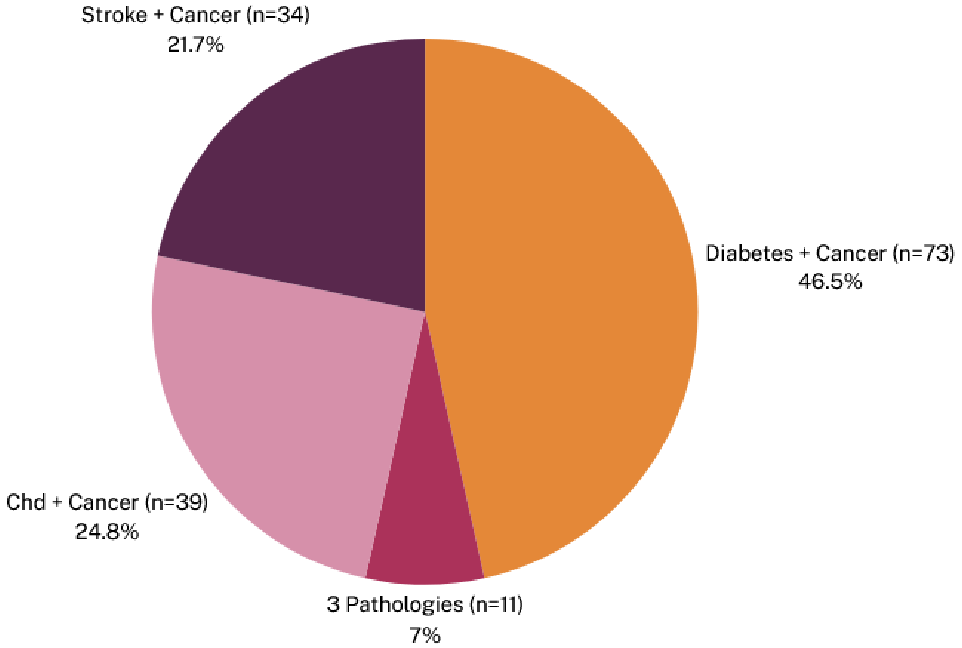

Supplementary Figure 16. Cluster 4, women sample.
Cancer: Other Cancers (157). CHD: coronary heart disease.

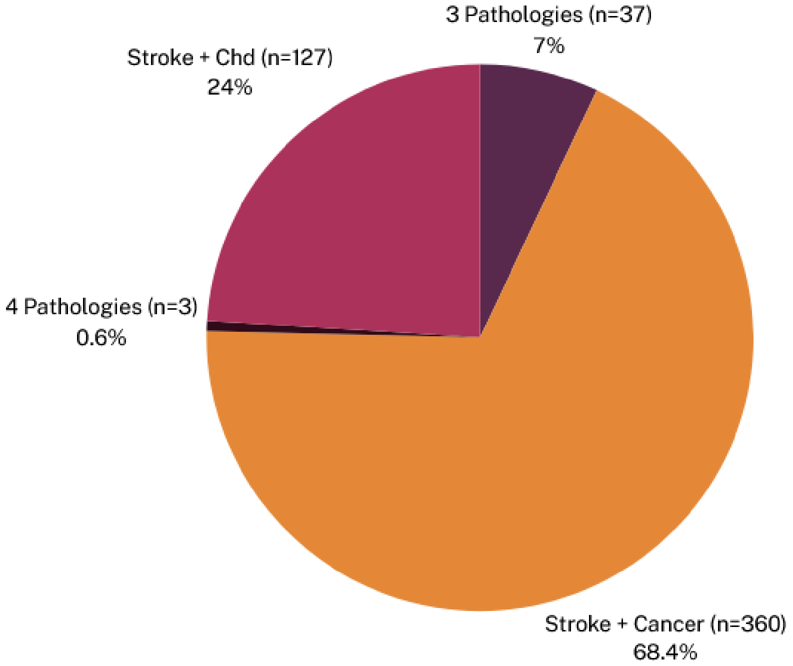

Supplementary Figure 17. Cluster 5, women sample.
Cancer: Breast cancer (113), Bodyof the uterus (25), Leukemia-Lymphoma (25), Liver-Pancreas (20), Ovary-Other female genitalia (19), Bladder-Renal (17), Digestive-Tract (13), Mouth-Larynx (3). CHD: coronary heart disease.

© 2024. Manfredi L. et al. Published online at http://www.aginganddisease.org/EN/10.14336/AD.2024.1166

SUPPLEMENTARY DATA

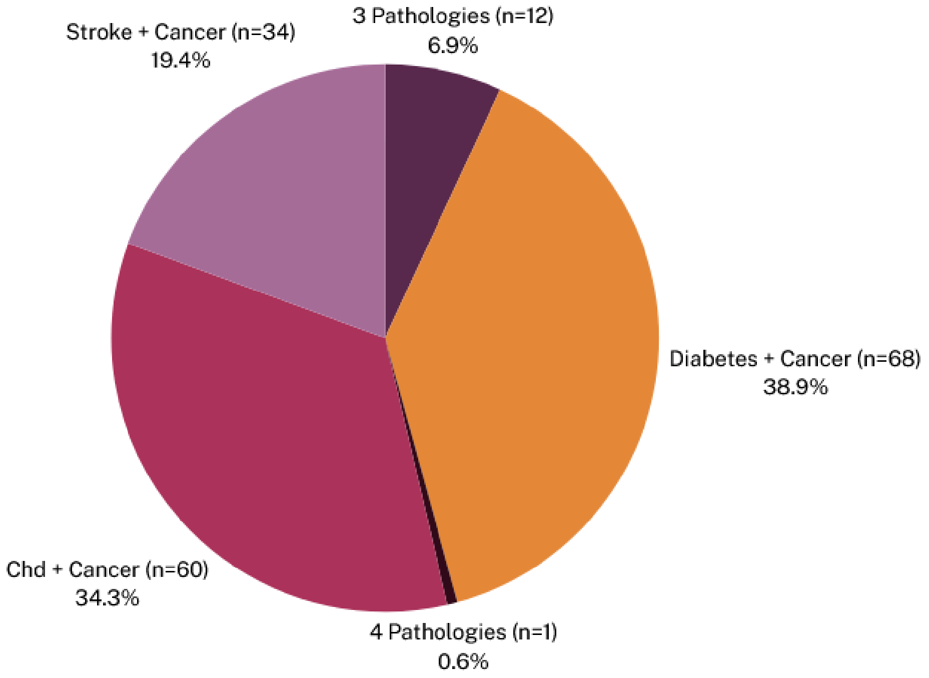

Supplementary Figure 18. Cluster 6, women sample.
Cancer: Melanoma (175)
CHD: coronary heart disease.

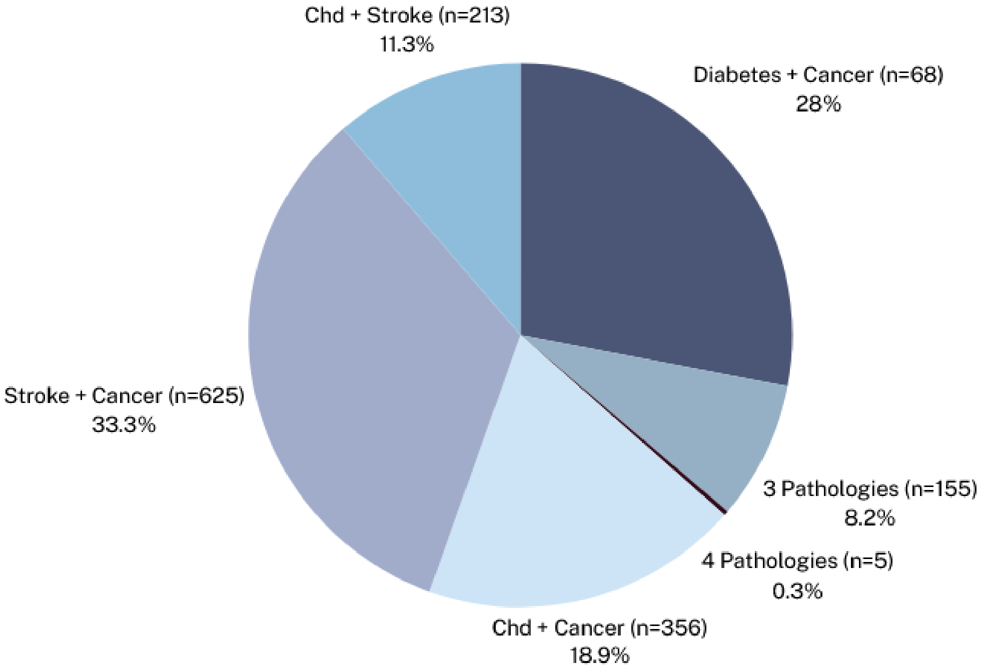

Supplementary Figure 19. Cluster 1, men sample.
Cancer: Prostate cancer (219), Melanoma (167), Lung cancer (155), Other Cancers (100), Leukemia-Lymphoma (90), Digestive-Tract (65), Mouth-Larynx (43), Liver-Pancreas (19)
CHD: coronary heart disease.

© 2024. Manfredi L. et al. Published online at http://www.aginganddisease.org/EN/10.14336/AD.2024.1166

SUPPLEMENTARY DATA

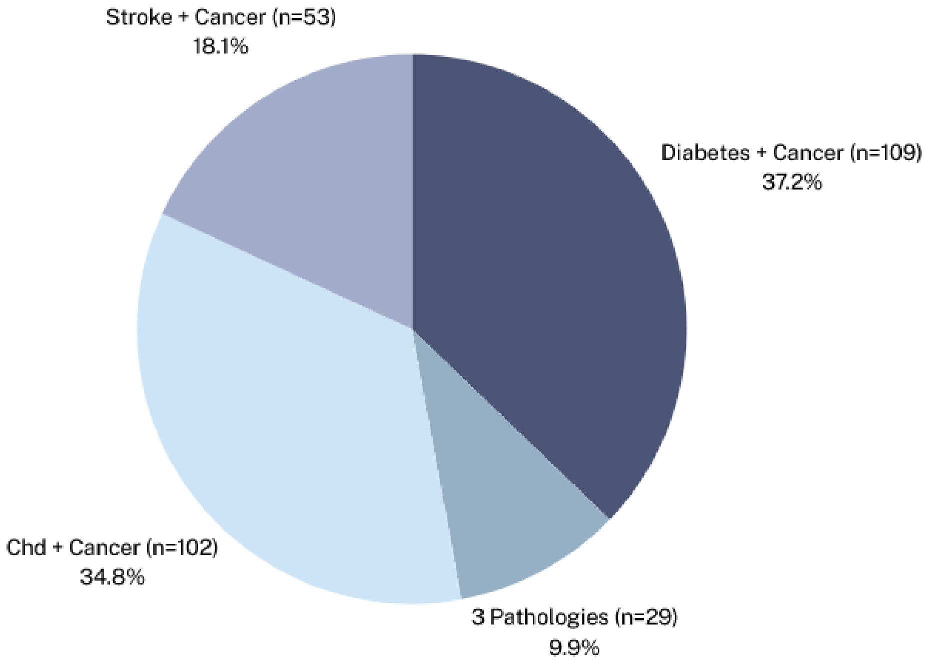

Supplementary Figure S20. Cluster 2, men sample**.**
Cancer: Bladder-Renal (293)
CHD: coronary heart disease.

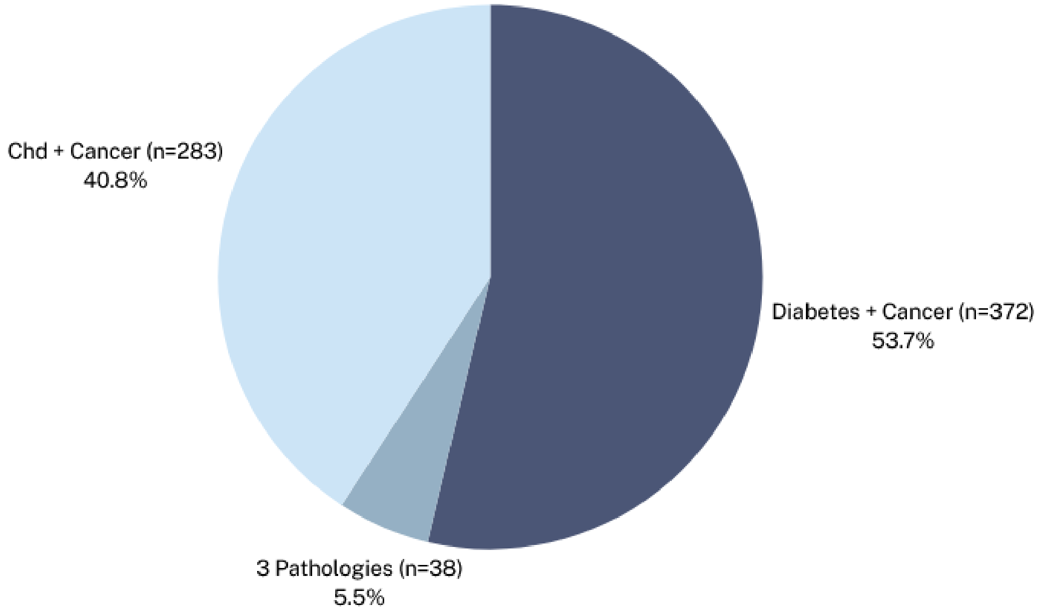

Supplementary Figure 21. Cluster 3, men sample
Cancer: Prostate Cancer (693)
CHD: coronary heart disease.

© 2024. Manfredi L. et al. Published online at http://www.aginganddisease.org/EN/10.14336/AD.2024.1166

SUPPLEMENTARY DATA

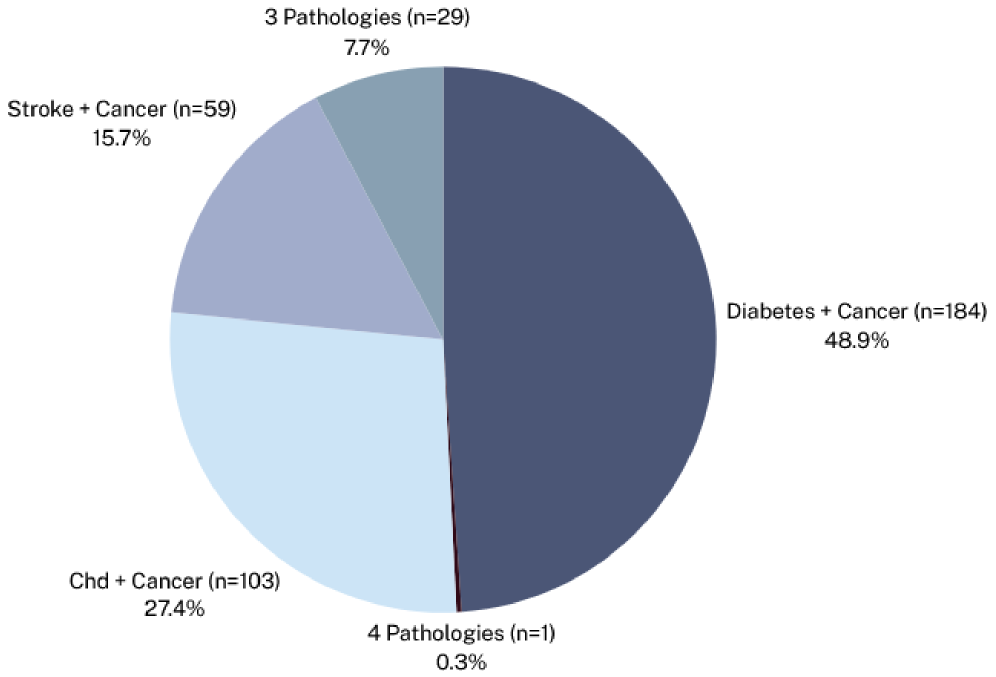

Supplementary Figure 22. Cluster 4, men sample.
Cancer: Colorectal Cancer (376)
CHD: coronary heart disease.

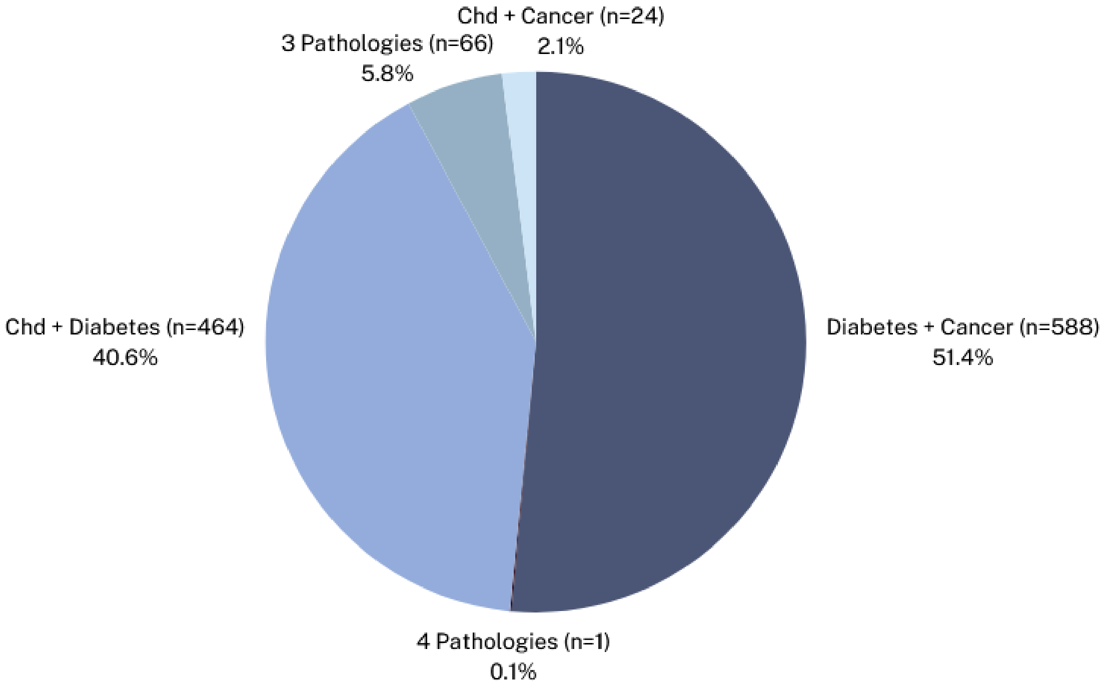

Supplementary Figure 23. Cluster 5, men sample.
Cancer: Liver-Pancreas (142), Melanoma (119), Lung cancer (113), Other Cancers (105), Leukemia-Lymphoma (97), Digestive-Tract (55), Mouth-Larynx (48)
CHD: coronary heart disease.

© 2024. Manfredi L. et al. Published online at http://www.aginganddisease.org/EN/10.14336/AD.2024.1166
